# Cryptococcosis in Colombia: Analysis of Data from Laboratory-Based Surveillance 2017–2024

**DOI:** 10.3390/jof12010067

**Published:** 2026-01-14

**Authors:** Jairo Lizarazo, Clara Inés Agudelo, Patricia Escandón, Elizabeth Castañeda

**Affiliations:** 1 Departamento de Medicina Interna, Hospital Universitario Erasmo Meoz, Cúcuta 540003, Norte de Santander, Colombia; jflizar@gmail.com; 2 Grupo de Microbiología, Instituto Nacional de Salud, Bogotá 111321, Colombia; cia1949@gmail.com (C.I.A.); ecastaneda21@gmail.com (E.C.)

**Keywords:** cryptococcosis, Colombia, epidemiology, *Cryptococcus neoformans*, *Cryptococcus gatti*, HIV, Cryptococcal meningitis, antifungal drug resistance

## Abstract

Since 1997, a laboratory-based survey on cryptococcosis has been conducted in Colombia. We present the results for the period 2017–2024. A total of 891 surveys were received. The overall incidence was 0.22 cases per 100,000 people. Among those living with HIV, the incidence was 38, and among HIV-negative people, it was 0.08. Cryptococcosis demonstrated a higher prevalence among men than women (3.2:1). Among patients living with Human Immunodeficiency Virus (HIV), the condition primarily affected younger adults (26–40 years). In contrast, among HIV-negative people, it was mostly observed in older adults (≥60 years). HIV infection was the most significant risk factor (63%), but another cause of immunosuppression was identified in 21.2% cases. Neurocryptococcosis was the most common form of presentation (62.2%), followed by disseminated cryptococcosis (31.1%). The diagnosis was confirmed by culture in 99.4% of patients; the most important sample was cerebrospinal fluid (67.3%), followed by blood (35.4%). *Cryptococcus neoformans* was identified in 93.1% of cases, and *Cryptococcus gatti* in 6.9%. Predominant molecular patterns were VNI (92.4%) and VGII (45.3%). The epidemiology of cryptococcosis in Colombia is changing, with a progressive decrease in HIV coinfection and an increase in other immunosuppressive conditions in older people. This study highlights the importance of cryptococcosis in Colombia and the need to report it in order to improve knowledge and thereby promote the quality of diagnosis and the opportunity for more effective treatment.

## 1. Introduction

In 2022, WHO published a list of priority pathogenic fungi, placing *C. neoformans* at the top of the critical priority group and *C. gattii* in the medium priority group. The document proposes strategies and actions focused on strengthening laboratory capacity, surveillance, investment in research and public health interventions [1].

Meningeal cryptococcosis remains the most common etiology of meningitis in adults living with HIV in sub-Saharan Africa, and an annual incidence of 152,000 cases of meningeal cryptococcosis resulting in 112,000 deaths has been estimated in several recent studies [2]. However, other risk groups such as solid organ transplant recipients, patients with malignancy and those receiving various types of immunosuppressive or biological treatments constitute an increasing proportion of reported cases in North America, Europe and Oceania [3,4,5,6].

Differences in patient characteristics and outcomes between patients living with HIV and HIV-negative patients have been described in several recent studies. Some of these studies have shown that HIV-negative patients receive delayed diagnoses and present higher mortality rates than patients living with HIV [4,5].

On the other hand, *C. neoformans* and *C. gattii* have different ecological niches [7] and present differences in epidemiology and clinical manifestations [8,9]. *C. neoformans* and *C. gattii* are distributed across different ecological niches. *C. neoformans* is ubiquitous and frequently found in pigeon excreta and decaying wood worldwide, while *C. gattii* is more prevalent in tropical and subtropical regions and is strongly associated with eucalyptus and other trees, although its range is expanding into temperate zones [7].

In Colombia, cryptococcosis has been studied since the middle of the last century, and numerous publications have described the disease (Table S1). A national survey on cryptococcosis has been conducted since 1997, which has provided information on the epidemiological, clinical and diagnostic characteristics of patients [10]. In this paper, the results of the surveys received between 2017–2024 are presented and includes a comparative analysis of populations according to the state of infection by HIV.

## 2. Materials and Methods

### 2.1. Study Design

We present a descriptive observational study in which a retrospective analysis of data gathered in the survey between 2017 and 2024 was performed. The survey was designed in 1997 and updated in 2022. Each format of the survey was previously filled out by health professionals attending the patient from public and private institutions, as well as from the public health laboratories of the Colombian political divisions (departments). The survey is divided into seven sections, which respectively hold the following information: (1) demographic data; (2) clinical manifestations; (3) risk factors; (4) laboratory diagnosis; (5) diagnostics images; (6) cryptococcosis classification; (7) treatment and other therapies (Table S2).

Complete surveys and isolates were sent to the Instituto Nacional de Salud in Bogotá, the study coordinating center, and a database was filled.

This study also took into account data collected over 28 years and divided it into four periods for analytical purposes: 1997–2003, 2004–2010, 2011–2016 [10] and 2017–2024 (this paper).

### 2.2. Case Definition

A case was defined as when a patient had clinical findings compatible with cryptococcosis together with one or more of the following criteria: isolation of the yeast from a normally sterile site, or from urine, sputum, bronchoalveolar lavage (BAL), skin lesions, and biopsies; direct visualization of blastoconidia with Indian ink in samples and visualization in biopsies and/or a capsular antigen by latex agglutination or Lateral Flow Analysis (LFA); and/or by DNA detection using the Film array in Cerebrospinal Fluid (CSF).

Disseminated cryptococcosis was defined by a positive culture from at least two different sites, including CNS, or a positive blood culture [11]. Relapses were considered to be when a patient presented with a new clinical episode of cryptococcosis six or more months after the initial diagnosis, with a positive culture.

### 2.3. Epidemiological Analysis

The number of cryptococcosis cases is given for each of the geographic departments (Colombian political divisions) that submitted information, and the prevalence is given for each of those according to the population projections reported by the Departamento Administrativo Nacional de Estadística (DANE) [12]. The mean cryptococcosis incidence per year in the general population was determined likewise, using as the denominator the average national population for the study period (year 2020). The incidence of cryptococcosis in the Acquired Immunodeficiency Syndrome (AIDS) population was obtained using as the denominator the average number of persons living with AIDS between 2017–2024 [13]. The incidence of cryptococcosis in the non-AIDS population was obtained using as the denominator the subtraction of the AIDS population from the total population. The frequency of each variable in the survey was determined, such as distribution by department, age groups, gender, and risk factors, among others.

### 2.4. Laboratory Tests, Antifungal Susceptibility Testing and Molecular Typing

Isolates submitted to the central laboratory were confirmed using conventional techniques [14], and species differentiation was performed by culturing the isolates in canavanine–glycine–bromothymol blue agar (CGB) [15]. Isolates were maintained as 10% glycerol stocks at −70 °C and in sterile distilled water at room temperature.

The susceptibility of isolates to six antifungal agents—amphotericin B (AMB), itraconazole (ITR), fluconazole (FLC), voriconazole (VRC), posaconazole (POS) and 5-flucyotsine (5FC)—was determined using methodology described by the Clinical and Laboratory Standards Institute [16] and/or the Sensititre Yeast One Plates (Thermo ScientificTM, Waltham, MA, USA) following the manufacturer’s instructions, with an inoculum set to a final concentration of 1.5–2.5 × 10^3^ cells/mL. The reference strains of *Candida krusei* ATCC 6258 and *Candida parapsilosis* ATCC 22019 were used as quality control strains.

The ranges of drug concentrations, tested by 2-fold serial dilutions, were as follows: 0.125–8 µg/mL for AMB, 0.015–16 µg/mL for ITR, 0.125–256 µg/mL for FLC, 0.008–8 µg/mL for VRC and POS and 0.06–64 µg/mL for 5FC. For the whole population for which the susceptibility testing was conducted, the frequency of MICs and the geometric mean MICs of each antifungal drug were determined. For the interpretation of antifungal susceptibility testing, Epidemiological Cutoff Values (ECVs) previously reported for *C.neoformans* and *C. gattii* were used. The MICs of antifungals, except AMB, were the lowest drug concentrations that produced ≥ 50% growth inhibition compared to the growth control; for AMB, MIC is considered as the lowest antifungal agent concentration that prevents any discernable growth [17,18].

The molecular pattern of isolates was determined by using Restriction Fragment Length Polymorphism (RFLP) of the *URA5* gene [19] with previous DNA extraction using the procedure described by Casali et al. [20].

### 2.5. Patients Living with HIV Versus HIV-Negative Patients

An additional analysis compared the survey results while taking into account the presence or absence of HIV infection in the patients.

### 2.6. Ethical Considerations

The present study has the endorsement of the ethics committee of the Instituto Nacional de Salud, and its implementation was subjected to the principles of medical research in human beings stated in the Helsinki Declaration. Although this survey is designed to be descriptive, patient identification is totally anonymized. No additional tests were demanded beyond those required by the consulting physician.

### 2.7. Statistical Analysis

Data were tabulated in Microsoft Excel^®^. For the numerical variables, the analysis was performed with measures of central tendency. For the categorical variables, the chi-square test or Fisher’s exact test was used, with a significance of less than 0.05 and 95% confidence, and the relative risk was calculated, for which the OpenEpi version 3.01 program was used [21].

## 3. Results

### 3.1. Survey Data

**Figure 1:**
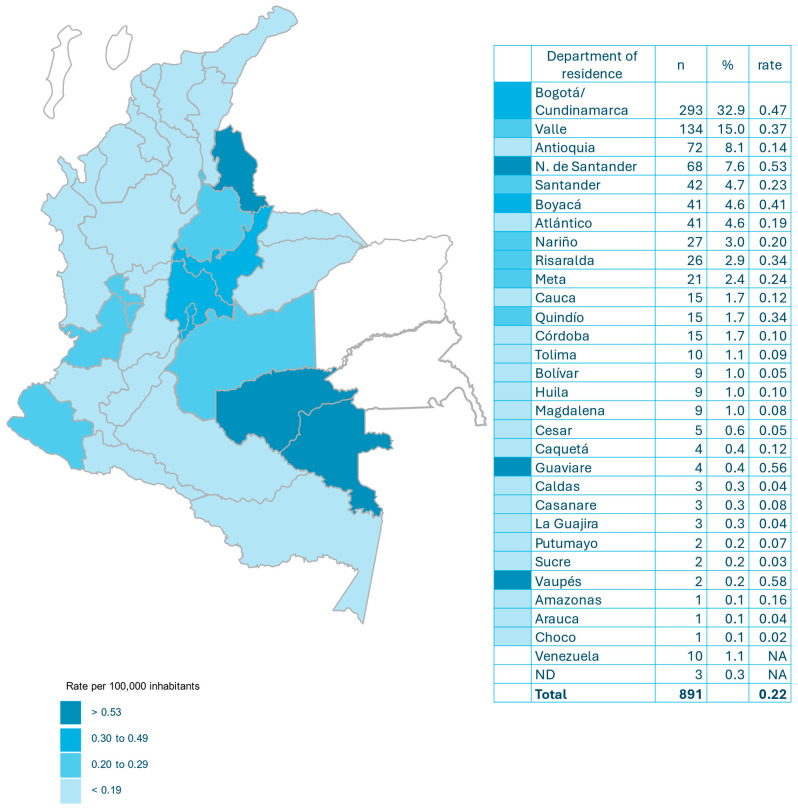
Incidence of cryptococcosis per 100,000 habitants and by department in Colombia (2017–2024).

A total of 891 surveys were obtained from 32 departments and the Capital District (Bogotá). The departments with the highest number of cases were as follows: Bogotá/Cundinamarca 293 (32.9%), Valle 134 (15.0%), Antioquia 72 (8.1%) and Norte de Santander 68 (7.6%). Thirty-four (3.8%) patients were originally from Venezuela (10 (1.1%) of them were residents of Colombia) (Figure 1).

### 3.2. Incidence

The annual incidence of cryptococcosis in Colombia was 0.22 per 100,000 inhabitants. The departments with the highest incidence were Vaupés 0.58, Guaviare 0.56, Norte de Santander 0.53, Bogotá/Cundinamarca 0.47 and Boyacá 0.41 (Figure 1).

### 3.3. Demographic Characteristics

Cryptococcosis was more common in men (76%) than in women (24%). The male-to-female ratio was 3.2:1. The mean age of the study population was 46.3 ± 17.9 years (median 43 years). The age groups most affected were 26–40 years (34.8%) and 41–59 years (31.0%). Only 1.5% of patients were pediatric individuals (Table 1).

### 3.4. Risk Factors

The most important risk factor was HIV infection (63.0%), followed by the use of corticosteroids or other immunosuppressive drugs (14.3%), chronic renal failure (8.0%) and diabetes mellitus (7.0%) (Table 1).

### 3.5. Clinical Manifestations

The most common symptoms and signs were headache (62.2%), fever (41.4%), mental confusion (39.8%) and nausea/vomiting (38.5%). Cryptococcosis of the central nervous system was the most common form of presentation (62.2%), followed by disseminated cryptococcosis (31.1%) and pulmonary cryptococcosis (5.1%) (Table 1).

### 3.6. Diagnostic Images

Chest X-rays were reported in 537 (60.3%) patients, and abnormalities were detected in 264 (49.2%), described as opacities (25.9%), pleural effusion (11.2%), nodules or masses (5.2%), cavitations (4.5%), calcifications (2.2%) or consolidations (1.7%). Cranial CT scans were performed in 436 (48.9%) patients, and the following abnormalities were described: cerebral atrophy (11.5%), infarcts (9.4%), masses (5.3%), hydrocephalus (6.0%), dilation of Virchow–Robin spaces (2.8%), cerebral edema (2.3%) and cerebritis (1.8%). A total of 206 (23.1%) brain Magnetic Resonance Images (MRI) were reported. The most frequent findings were infarction (14.6%), hydrocephalus (12.6%), cerebritis (7.3%), cerebral atrophy (6.8%), masses (4.8%), meningitis (4.4%), the dilation of Virchow–Robin spaces (3.9%) and cerebral edema (1.5%).

### 3.7. Treatment

Antifungal induction therapy was reported in 509 cases (57.1%): 455 (89.4%) of these cases received AMB, with 117 (23%) taking AMB alone and 338 (66.4%) taking it in combination with another antifungal agent. FCZ and 5FC were reported in 54 cases (Table 1).

### 3.8. Mortality and Relapses

Mortality was reported in 181 (20.3%) patients. There were 29 (3.2%) relapses (Table 1).

### 3.9. Mycological Diagnosis of Cryptococcosis

Overall, diagnoses of cryptococcosis were determined by culture, direct examination, antigenemia and Film Array. In 886 (99.4%) of the cases, culture was the diagnostic method for cryptococcosis; direct examination yielded positivity in 472 (53.0%) samples; and samples tested by antigenemia were reactive in 318 (35.7%) patients (Table 2).

From the 886 (99.4%) cases diagnosed by culture, 595 (67.3%) isolates were recovered from CSF, 313 (35.4%) from blood, 29 (3.3%) from BAL and 17 (1.9%) from biopsies, other samples, 11 and 12 without data. Detailed diagnostic methods and samples type are described in Table S3.

Regarding the complex species involved with cryptococcosis, the fungus was recovered from 886 samples; most cases were produced by *C. neoformans* in 779 (93.1%) samples, whilst in a lower proportion, *C. gattii* was identified in 58 (6.9%). In 49 cases, the isolates were non-viable.

Molecular pattern was determined in 316 isolates (262 *C. neoformans* and 54 *C. gattii*). VNI was identified as the most prevalent molecular pattern in *C. neoformans* (242 (92.4%) isolates). In *C. gattii*, VGII was the most prevalent (25 (46.3%) isolates) (Table 2).

### 3.10. Antifungal Susceptibility

The susceptibility to antifungal drugs was determined in 180 *C. neoformans* isolates and in 40 *C. gattii* isolates. In the absence of clinical breakpoints established for *Cryptococcus* according to CLSI, the MIC values for each antifungal drug tested were considered to determine whether an isolate was wild-type (WT) according to the ECVs (Table S4) [17,18].

WT isolates for AMB were identified for 90% *C. neoformans* isolates and 80% *C. gattii*. For ITZ, the MIC values for *C. neoformans* and *C. gattii* were considered to correspond to the WT in 85.5% and 77.5% of isolates, respectively. Interestingly, a high percentage of isolates, both *C. neoformans* and *C. gattii*, were classified as non-wild-type (NWT) for FLC, VRC and POS. Within this context, 84.5% of *C. neoformans* isolates were NWT for FLC, and all *C. gattii* isolates. As for 5-FC, 16.7% of *C. neoformans* isolates were classified as NWT, and 62.5% of *C. gattii* isolates as well. All except two *C. neoformans* isolates showed a non-wild-type pattern for at least one antifungal agent (Table S4). In general, there are no genotype-dependent differences in MIC values for *C. gattii* isolates.

### 3.11. Characteristics in Patients Living with HIV and HIV-Negative Patients

*HIV infection as a risk factor*. This report shows a downward trend in HIV infection among the population with cryptococcosis. The current rate of 63% is the lowest recorded in the 28 years that the survey has been conducted, with a corresponding increase in the HIV-negative population (Figure S2).

*Incidence*. The annual incidence of cryptococcosis in patients living with HIV was 38 per 100,000 inhabitants. The departments with the highest incidence were Vaupés (595), Boyacá (184) and Norte de Santander (116). In the HIV-negative population, the annual incidence was 0.08/100,000 and was highest in the departments of Guaviare (0.44), Vaupés (0.28) and Boyacá (0.19). The ratio between the incidence of cryptococcosis in patients living with HIV and HIV-negative patients in Colombia was 475. The departments with the highest rates were Magdalena (2400), Vaupés (2125) and Huila (1450) (Table S5).

*Demographic characteristics*. There was a higher prevalence of males among patients living with HIV (4.5:1.0) compared to HIV-negative patients (1.9:1:0) (Table 3).

While in patients living with HIV, the age groups most affected by cryptococcosis were those aged 26–40 years (45.8%) and 41–59 years (32.3%); in HIV-negative patients, the groups aged ≥ 60 years (48.5%) and 41–59 years (28.8%) predominated. In both groups, patients ≤ 16 years of age were rare—0.9% in patients living with HIV and 2.4% in HIV-negative patients—therefore it is interpreted that the two groups are statistically different in age distribution, with HIV-negative individuals typically being older. (Table 3, Figure S2a,b). In 176 patients (31.4%) with HIV, cryptococcosis defined AIDS.

*Risk factors*. Other risk factors were reported in patients living with HIV, the most common being the use of corticosteroids or other immunosuppressive drugs (8.6%), chronic renal failure (2.1%) and diabetes mellitus (2.0%). In the HIV-negative population, these same risk factors predominated but in a higher proportion (use of corticosteroids or other immunosuppressive drugs (23.9%), chronic renal failure (17.9%) and diabetes mellitus (15.5%) (Table 3).

Fourteen patients were diagnosed with COVID-19, three (21.4%) of whom had an HIV infection.

*Clinical forms*. CNS involvement was the most common clinical manifestation in both groups but was more notable in patients living with HIV (69.5% vs. 49.7%) (*p* < 0.0001, risk ratio 1.387, CI 1.233, 1.561). On the other hand, disseminated and pulmonary forms were predominantly seen in HIV-negative patients (36.4% and 10.9%, respectively) compared to patients living with HIV (28.0% and 1.8%, respectively) (Table 3).

*Symptoms and signs.* Patients living with HIV had more frequent clinical manifestations than HIV-negative patients, especially headache, nausea and vomiting. (Table 3).

*Diagnostic images***.** Chest X-ray abnormalities were observed more frequently in HIV-negative patients (58% vs. 44.7%). In patients living with HIV, the most frequently reported abnormalities were opacities (25.6%), pleural effusion (5.6%), nodules or masses (5.3%), cavitations (5.9%), calcifications (2%) and consolidations (1.7%). HIV-negative patients presented the following abnormalities: opacities (26.5%), pleural effusion (22.1%), nodules or masses (5%), cavitations (1.7%), calcifications (2.8%) and consolidations (1.7%).

The abnormalities described in the cranial CT scan according to HIV-positive or HIV-negative status were as follows: cerebral atrophy (10.5% vs. 11.5%), cerebral infarcts (8.6% vs. 11.5%), hydrocephalus (4.1% vs. 10.7%), masses (4.5% vs. 7.4%), the dilation of Virchow–Robin spaces (3.5% vs. 0.8%), cerebral edema (1.9% vs. 3.3%) and cerebritis (1.6% vs. 2.5%).

In brain MRIs, depending on HIV status (positive or negative), the following abnormalities were reported: cerebral infarcts (18.4% vs. 7.1%), hydrocephalus (10.3% vs. 17.1%), cerebral atrophy (7.4% vs. 4.3%), cerebritis (6.6% vs. 8.6%), masses (3.7% vs. 7.1%), the dilation of Virchow–Robin spaces (5.9% vs. 0%), meningitis (2.2% vs. 5.7%) and cerebral edema (1.5% vs. 2.9%).

*Treatment***.** Antifungal treatment was similar in both groups. It was based on the use of amphotericin B either alone (20.7% in patients HIV+, 28.5% in HIV-negative patients) or in different combinations with flucytosine, fluconazole and itraconazole (70.1% vs. 67.0%) (Table 3).

*Mortality and relapses*. In patients living with HIV+, mortality was 19.4%, while in HIV-negative patients, it was 21.8%. There were 27 (3.0%) relapses in HIV-positive patients and 2 (2.2%) in HIV-negative patients (Table 3).

In persons living with HIV, the most frequent species recovered was *C. neoformans* (97.1%), molecular type VNI (90.8%); in HIV-negative patients, despite *C. neoformans* being the predominant species (93.1%) with molecular type VNI (92.4%), *C. gattii* accounts for 6.9% of the cases of cryptococcosis in this population (Table 3).

## 4. Discussion

Cryptococcosis continues to be a significant mycosis in Colombia, as demonstrated by the results of this survey covering almost three decades of national surveillance [10]. Most surveys continue to come from four departments (Bogotá/Cundinamarca, Valle, Antioquia and Norte de Santander), which account for 63.6% of cases and are where 47.1% of the Colombian population lives. Nevertheless, in the latest period, Santander, Boyacá and Atlántico have made a significant contribution to the number of cases (13.9%). The passive nature of the survey does not favor information gathering; however, cases have been reported in Vaupés and Guaviare, departments that were not previously included [10]. Also noteworthy is the emergence of Venezuelan patients, some residing in Colombia and others crossing the border in search of health services that are lacking in their country of origin [22] (Figure 1, Table S6).

The incidence of cryptococcosis in Colombia has remained virtually unchanged in recent years: 0.22 per 100,000 inhabitants compared to 0.23 per 100,000 inhabitants [10]. Currently, Vaupés and Guaviare have the highest incidence, but Norte de Santander and Bogotá/Cundinamarca continue to have incidence rates above the national average (Figure 1). Globally, the incidence of meningeal cryptococcosis was 193,000 cases in 2020 [23]. In the United States, the incidence of cryptococcosis has decreased by approximately 50% [3]. Also, in France, the incidence has fallen from >0.5/100,000 to 0.2/100,000 during this century [24,25]. In the Brazilian Amazon, an area with a high risk of cryptococcosis, the annual incidence of the disease ranged from 0.26/100,000 to 0.40/100,000 from 2008 to 2018 [26]. In Botswana, a sub-Saharan African country, with a high annual incidence of meningeal cryptococcosis, the rate has decreased from 15.0/100,000 to 7.4/100,000 (50.6%) over a period of 10 years [27] The decrease in the global incidence of cryptococcosis can be attributed to the enhanced management of HIV infections [23]. In Taiwan, the incidence of cryptococcosis increased from 1.48 in 2002 to 2.76 in 2015, marked by an increase in the incidence of non-meningeal forms of cryptococcosis (from 0.86 to 2.12) and HIV-negative patients [28]. A recent publication [6] has documented a notable rise in the prevalence of cryptococcosis among patients who are HIV-negative in Australia and New Zealand.

The incidence of cryptococcosis has decreased by 65% in our population living with HIV (110/100,000 to 38/100,000) [10] (Table S5). In Colombia, cryptococcosis is almost 500 times more common in people living with HIV (Table S5). This fact is likely to contribute to the difficulty in diagnosing cryptococcosis in HIV-negative patients because clinicians do not consider it, especially in individuals without apparent immunosuppression. The overall incidence of meningeal cryptococcosis in patients living with HIV decreased by 32% between 2014 and 2020 [2]. In 2020, it was estimated at 152,000 cases; for the HIV-negative population, the annual incidence was 41,000 cases, half of which had no known immunodeficiency [23]. In Latin America, there were an estimated 12,000 cases of HIV-associated meningeal cryptococcosis [2]. Despite advances in antiretroviral therapy treatment in sub-Saharan Africa, the prevalence of cryptococcosis in patients living with HIV is high (8.3%) [29,30].

Cryptococcosis mainly affects males [3,4,6,27,31,32,33,34]. The predominance of males has been slowly declining among Colombian patients, reflecting the decrease in the population living with HIV. There has been a higher prevalence of males in the population living with HIV [4,6,27,35,36]. This male dominance in these patients has also been declining in our survey; in the early years of the survey, it was 7.1:1, while in this study, it was 4.5:1 (Table S7, Table 1 and Table 3). In an extensive series of cases in Argentina (75% living with HIV), the ratio of 3:1 in favor of males remained unchanged over a period of 30 years [36]. In some cases, a female predominance has been documented, as seen in the Democratic Republic of the Congo (51.7%) [37] and Uganda (53–61%) [38]. The underlying reasons for the predominance of cryptococcosis in males remain to be fully elucidated. A body of evidence suggests that the immune response in males may be less effective in controlling infection by *C. neoformans* [39].

Cryptococcosis has historically been a disease that has affected young patients living with HIV [33]. In this survey, 78.1% of patients living with HIV were between 26 and 59 years old. In contrast, 77.3% of HIV-negative patients were over 40 years old (Table 3). This is similar to what has been reported in China, where 70.3% of patients were 40 years old or older [34]. In Japan, the median age of a series of cryptococcosis cases in HIV-negative patients was 71 years [5]. In France, the median age was 61 years [40]. In Australia and New Zealand, the mean was 58.3 years [6], and in the United States, it was 58 years [3]. According to published data, cryptococcosis rarely affects children, both in the population living with HIV and in HIV-negative individuals. The epidemiological and clinical characteristics of pediatric cryptococcosis are similar to those in adults (Table 3) [41].

In recent years, our survey has shown a decrease in the prevalence of HIV infection as a risk factor. Although HIV infection is the primary risk factor for cryptococcosis in our population (63%), the presence of additional risk factors associated with immunosuppression has been observed in 21.2% of patients, exhibiting an upward trend in our survey. These factors are also present, albeit less frequently, in patients living with HIV (Figure S1, Table 1, Table 3 and Table S8).

HIV infection is the primary risk factor for cryptococcosis, particularly in Africa, which has the highest number of reported cases (53.9% of all meningeal cryptococcosis cases) [2]. In sub-Saharan Africa, nearly all patients with cryptococcosis are HIV-positive (97.6% in the Democratic Republic of Congo and 96% in Uganda, for example) [37,42]. In Thailand (90.6%) [33] and Brazil (79.2%) [32], the prevalence of HIV infection remains high. In other regions of the world, particularly in countries with greater economic resources, the prevalence of HIV infection is significantly lower. According to George [3], the United States has a 56.1% rate, while France has a rate of 42.4% [4]. In addition, China has a rate of 30.6% [43], Japan 12.0% [5], Australia and New Zealand 10.3% [6], and Taiwan 6.1% [28]. In these countries, however, other factors have assumed greater importance. These factors include immunosuppressive therapies, solid organ transplantation, malignancies, autoimmune disorders and other diseases that affect immunity, such as diabetes, chronic renal failure and cirrhosis [4,5,31]. Subtle immune defects are emerging among apparently immunocompetent patients, as evidenced by the presence of anti-granulocyte-macrophage colony-stimulating factor (GM-CSF) autoantibodies. These autoantibodies predispose individuals to cryptococcosis [44,45].

Cryptococcosis associated with COVID-19 has been reported but is not as common as other mycoses. Few of these patients have underlying immunosuppression [46]. Patients living with HIV are likely to have higher mortality [47].

Neurocryptococcosis was the most prevalent form of this mycosis, with a higher incidence among individuals living with HIV. Globally, meningeal cryptococcosis is the most common form of this mycosis [2]. The greater meningeal involvement observed in our patients living with HIV has been observed in other studies [3,4]. Conversely, the higher frequency of pulmonary cryptococcosis observed in HIV-negative patients has been documented in several studies [3,4,6]. The diagnosis of disseminated forms has increased in Colombia from 13.3% to 31.1% [10]. This increase occurred in both patients living with HIV and HIV-negative patients (Table 3) and is attributed to the higher number of blood cultures performed. In this analysis, positive blood cultures were 35.4% (Table 1 and Table 2).

As shown in Table 3, all clinical manifestations were more prevalent among patients living with HIV. The differences in headache and nausea/vomiting were particularly significant, correlating with a higher incidence of intracranial hypertension in patients living with HIV.

Almost half of the patients analyzed had abnormalities in their chest X-rays, the most common being opacities and pleural effusion. The manifestations of pulmonary cryptococcosis are diverse and range from an isolated pulmonary mass to multiple pulmonary nodules or a generalized disseminated interstitial infection [48]. Not all patients with cryptococcal meningoencephalitis have abnormal CNS imaging (47% of CT scans and 8% of MRIs are normal). Brain MRI is the test of choice due to its superior diagnostic sensitivity. The abnormalities found in this study are those classically described: pseudocysts or cryptococcomas, the dilation of Virchow–Robin spaces, signs of meningitis, cerebral edema and ischemic lesions [49,50]. A recent Brazilian study [51] found no significant differences in mortality related to different radiological patterns. The presence of pseudocysts was associated with the need for hospital readmission, and ventricular shunting was significantly associated with pseudocysts and hydrocephalus.

Antifungal induction therapy was based on amphotericin B alone or in different combinations according to international management guidelines [52,53,54,55]. In recent years, Colombia has seen increased access to other forms of amphotericin B, such as liposomal and lipid complex. Flucytosine has also recently become available. The most effective induction treatment for meningeal cryptococcosis, the most common and severe form of the disease, is a combination of amphotericin B and flucytosine [1,55].

The hospital mortality rate of 20.3% described is lower than the previously reported rate of 47.5% [10] and probably reflects earlier diagnosis and access to flucytosine in recent years. It has been estimated that 112,000 people living with HIV die each year from meningeal cryptococcosis, and this mycosis is responsible for 19% of AIDS-related mortality [2]. In countries with greater resources, one-year mortality rates of 21% have been reported in Australia [6], 30% in the United States [31], and 22% at 30 days in France [4]. In Brazil, hospital mortality from cryptococcosis was estimated at 29% [32]. The mortality rate for meningeal cryptococcosis depends on how early it is diagnosed and the type of antifungal therapy available. Other factors that influence mortality include the control of intracranial hypertension, the timing of antiretroviral therapy initiation and adherence to treatment [23]. In general, the crude mortality rate for meningeal cryptococcosis is assumed to be 60% [23], although the figure varies significantly (20–70%) depending on the country’s level of development [56,57]. There was no significant difference in mortality between patients living with HIV and HIV-negative patients (Table 1). There have been recent reports of higher mortality in HIV-negative patients [3,4,35], likely due to delayed diagnoses and the patients’ underlying pathologies.

Although the diagnosis of cryptococcosis may be challenging, as it involves a combination of symptoms (headache, fever, mental alterations, seizures, etc.) confirmed by laboratory testing, culture of the fungus remains the gold standard for the diagnosis of cryptococcosis. Colombia is not an exception to this practice, since 99.3% of our patients reported through the survey were identified by culture as harboring the infection, specially from CSF samples, but it is important to call out the attention on the number of blood samples in which the infection was diagnosed, meaning a dissemination of the pathogen in these patients. (Tables S3 and S9). Together with Indian ink examination, these two diagnostic tests are an essential tool to detect the fungus in patient samples. Despite culture being the benchmark for diagnosis, growth can take up to seven days, and sensitivity can be as low as 82.4%, limited by false negatives in patients with a low fungal burden [58].

The detection of the cryptococcal antigen by antigenemia has been reported to have sensitivity between 99.1% and 100% and a specificity of 100%, therefore being a useful tool for the diagnosis of this infection. This has been confirmed by the fact that WHO has recommended Cryptococcal Antigeg (CrAg) as the first-line diagnostic test for cryptococcosis, emerging as the new gold standard for detecting cryptococcal meningitis in patients living with HIV [54]. Unfortunately, our analysis revealed that this test is being underused, as only 35.7% of the surveys reported the use of these tests, despite the demonstration of their utility (Table S3). In 2022, a cross-sectional study across 14 countries showed that 40% of patients had no access to CrAg testing, while only 25% had routine access to CrAg [59]; furthermore, a modeling study outside Africa suggests that most countries outside Africa do not have national CrAg screening programs, with some exceptions such as Guatemala [2]. In a previous study conducted in Africa, which enrolled 832 persons with suspected meningitis and had 666 CSF samples tested, CrAg LFA and CrAg Latex had a sensitivity of 99.3% and 97.8%, specificity of 99.1% and 85.9%, positive predictive values of 99.5% and 92.6% and negative predictive values of 98.7% and 95.5%, respectively, for the diagnosis of cryptococcal meningitis [60]. These data, together with the incidence of cryptococcosis reported in the present study, should call the attention of public health authorities in Colombia of the exceptional predictive value of CrAg testing and the need to expand this tool in screening programs aimed at identifying patients with cryptococcal antigenemia, who are at risk for developing cryptococcal meningitis, together with the fact that CrAg has transformed the diagnosis of HIV associated cryptococcal meningitis, resulting in shorter induction regimens with amphotericin B treatments in combination with flucytosine and fluconazole, which translates into better survival times.

Overall, according to the global guideline for the diagnosis and management of cryptococcosis published in 2024, recommendations for diagnosis in patients with suspected or confirmed cryptococcosis require medical review for central nervous system, pulmonary or any other body site involvement, which includes lumbar puncture with measurements of CSF opening pressure, glucose, protein and cell counts, microscopy, culture and CrAg quantification; the tittering of blood CrAg and cultures of blood, sputum (or other respiratory specimens) and/or of other affected body sites; and brain and chest imaging [55].

As for the species of *Cryptococcus* identified in Colombian patients, *C. neoformans* is the most prevalent cause of cryptococcosis in 88.0% of the cases, in which molecular type VNI was the most frequent pattern identified. Our findings align with the reports of the distribution of molecular types in Latin American isolates of *Cryptococus*, where 49.3% of the typed strains belong to VNI [61], with a conserved pattern of distribution in Colombia across time, where VNI has remained the most prevalent molecular pattern (49.4%) in clinical isolates of *C. neoformans* [19]. Likewise, for *C. gattii* isolates typed, VGII continues to be the most prevalent pattern in Colombian strains, which is in agreement with the data reported in the region, with 69.4% of the clinical *gattii* isolates belonging to VGII molecular type [61].

Antifungal susceptibility testing plays an important role in detecting *in vitro* resistance, especially in those scenarios where a patient is not responding to therapy. This *in vitro* resistance is translated in breakpoint (BP) values, but the Clinical Laboratory Standards Institute (CLSI) BP values are not available for *Cryptococcus* spp. However, some authors have reported that MIC values of >16 g/mL for azoles in *C. neoformans* may be predictive of clinical failure [62]. Due to the lack of BP for the most common antifungal agents, isolates can be classified by means of ECV/ECOFF as being mutant (NWT, which could be more difficult to treat) or WT, which is not the same as resistant or susceptible [16] CLSI, 2022.

This new concern of antifungal resistance in *Cryptococcus*, specially to fluconazol, that is emerging alongside an increase in antimicrobial resistance burden, is seen in our study, where 84.5% of the *C. neoformans* isolates tested had MIC values above 16 g/mL (Table S4). This resistance, whether primary or secondary, can result in delayed fungal clearance and may be associated with an increased incidence of relapse [63], especially in countries where the need to switch to other treatments is often unachievable due to high cost and where there is a limited availability of second-line antifungals such as itraconazole, voriconazole or posaconazole [64] and difficulty in administering weekly amphotericin B as a maintenance treatment. Non-wild-type *C. gattii* isolates’ reactions to fluconazole are of great concern in our study, since all the isolates tested for antifungal susceptibility (*n* = 40) yielded MIC values ≥ 8 g/mL, similar to the findings in countries like the USA, where high MIC values were reported in a large set of strains recovered across the country [65].

For amphotericin B, low resistance rates (non-wild-type MIC) have been reported in the few studies found in the literature; in 2013, Andrade-Silva et al. reported resistance rates in Brazil of 11% based on 95 isolates from HIV patients [66], and as low as 2% resistance rate in the Indian population [67]. Espinel-Ingroff A et al. reported in 2012 that the rates of non-WT amphotericin B MICs were very similar between both species of *Cryptococcus* (4.3% and 4.1%, respectively) [18]; in our study, 10% of the *C. neoformans* isolates analyzed had MICs that fell in the non-WT category, whilst for the *C. gattii* Colombian isolates tested, this rate increased to 20% (Table S4). Overall, cryptococcal infections caused by these two species are clinically similar; however, it is well documented that infections caused by *C. gattii* have delayed treatment responses and other complications when compared to cryptococcosis caused by *C. neoformans* [52,68]. As for flucytosine, 16.7% and 62.5% of the *C. neoformans* and *C. gattii* isolates were classified as non-WT, respectively; this is rather important, since higher rates of strains were classified in this category when compared to the same study by Espinel-Ingroff A, et al. in 2012 [17] that included a set of isolates from different countries, in which non-WT MICs were lower among *C. neoformans* (4.3%) and *C. gattii* (10.8%), respectively (Table S4).

These results highlight the need to routinely perform antifungal susceptibility testing in cryptococcal isolates to detect resistant strains, whose patterns are suggested to be species-specific.

Given the significant global burden of cryptococcosis, high mortality in real-world settings, significant neurological sequelae and resistance to fluconazole, a recent meta-analysis calls the WHO’s attention to the need to increase surveillance of the disease and its outcomes, long-term disability and susceptibility to antifungal drugs [69].

Among the strengths of this data are the epidemiological characterization of patients, the large number of isolates and their typing, and the determination of antifungal resistance. However, there are also limitations to consider, such as the non-mandatory nature of case reporting, which currently leads to the underreporting of cryptococcosis. This situation is expected to improve with the new legal provision requiring mandatory reporting [70]. Our limitations are several: due to logistical constraints, it was not feasible to conduct the molecular typing of a larger number of *C. neoformans* isolates. The survey results are an initial approximation of patients hospitalized for cryptococcosis. The study does not include follow-ups with patients, and the mortality data only applies to the initial hospitalization. The study does not address sequelae or surgical treatments, nor does it report the occurrence of immune reconstitution syndromes. Future work in Colombia should be focused on strengthening surveillance, with emphasis on the complete filling of the survey and the submission of isolates to the National Reference Laboratory of the INS; the mandatory determination of MICs and molecular patterns of the received isolates by the National Reference Center at the INS; and a patient tracking system in participating centers that report the highest number of isolates, highlighting the need for determining the MICs of the isolates.

## 5. Conclusions

The epidemiology of cryptococcosis is changing worldwide. It is important to note that the disease is no longer exclusively affecting young people living with HIV. Instead, it is becoming a mycosis that is affecting older people with various conditions that cause immunodeficiency. Healthcare professionals should be aware of these changes and consider this diagnosis in patients with meningoencephalitis and pulmonary masses.

## Figures and Tables

**Table 1:** Distribution of patients with cryptococcosis in Colombia (2017–2024).

Patients‘ Epidemiological and Clinical Characteristics	Total	
	*n*	%
**Sex**		
Male	677	76.0
Female	214	24.0
**Total**	**891**	**100.0**
Male: female ratio: 3.2:1		
**Age group**		
≤16	13	1.5
17–25	81	9.1
26–40	310	34.8
41–59	276	31.0
≥60	211	23.7
**Total**	**891**	**100.0**
Risk factor *		
AIDS	561	63.0
Corticosteroids/other immunosuppressive drugs	127	14.3
Chronic renal failure	71	8.0
Diabetes	62	7.0
Autoimmune disease	50	5.6
Solid tumor	44	4.9
Transplantation	28	3.1
Cirrhosis	20	2.2
Hematologic malignancies	18	2.0
Others **	4	0.4
Unknown or no risk factors	141	15.8
**Symptoms/signs**		
Headache	554	62.2
Fever	369	41.4
Confusion	355	39.8
Nausea/vomiting	343	38.5
Cough	188	21.1
Intracranial hypertension without hydrocephalus	156	17.5
Seizures	135	15.2
Neck stiffness/meningeal signs	106	11.9
Others ***	273	30.6
**Clinical presentation**		
CNS	554	62.2
Disseminated	277	31.1
Pulmonary	46	5.1
Other ****	14	1.6
**Total**	**891**	**100.0**
**Treatment**		
AmB alone	117	23.0
AmB combined	338	66.4
Others *****	54	10.6
**Total**	**509**	**57.1**
**Mortality**	181	20.3
**Relapses**	29	0.2

* Risk factor: A patient may have more than one risk factor. ** Risk factors: idiopathic CD4 lymphocytopenia (2), sarcoidosis (1), alcoholism (1). *** Symptoms/signs: abnormal vision (76), double vision (60), meningeal signs (48), focal neurological deficit (45), hydrocephalus (44). **** Clinical presentation: cutaneous (2), skeletal (1), lymph node (1), peritoneal (1), unspecified (9.). ***** Treatment: fluconazole (52), flucytosine (2).

**Table 2:** Laboratory diagnosis of cryptococcosis, sample type, and complex identification, Colombia (2017–2024) (*n* = 891).

Laboratory Data	*n*	%
**Diagnostic method**		
Culture	886	99.4
Direct examination	472	53.0
Antigenemia (Latex, Lateral Flow Analysis (LFA)) *	318	35.7
Film array **	33	3.7
**Sample type**		
CSF	596	67.3
Blood	313	35.4
BAL	29	3.3
Biopsy (lung, skin, others) ***	17	1.9
Other sterile body fluids	8	0.9
Catheter	2	0.2
Tracheal aspirate	1	0.1
ND	12	1.4
Species complexes ****		
*C. neoformans*	779	93.1
*C. gattii*	58	6.9
**Total**	**837**	**100.0**
**Molecular patterm**		
VNI	242	92.4
VNII	17	6.5
VNIII	3	1.1
VNIV	0	0
Total	**262**	**100.0**
VGI	19	35.2
VGII	25	46.3
VGIII	10	18.5
VGIV	0	0
**Total**	**54**	**100.0**

* Latex (292), LFA (26). ** Diagnosis by Film Array only in 5 patients. *** Biopsies: lung (11), brain (3), skin (1), lymph node (1), ND (1). **** Isolates non-viable (49).

**Table 3:** Characteristics of cryptococcosis patients according to infection with HIV (2017–2024).

Variable	HIV + N = 561 (63.0)		HIV − N = 330 (27.0)		Total N = 891		*p*
	*n*	%	*n*	%	*n*	%	
**Sex**							
Male	459	81.8	218	66.1	677	76.0	<0.0001
Female	102	18.2	112	33.9	214	24.0	
Male: female ratio	4.5:1.0		1.9:1.0		3.2:1.0		
**Age group in years**							
≤16	5	0.9	8	2.4	13	1.5	<0.0001
17–25	67	11.9	14	4.2	81	9.1	
26–40	257	45.8	53	16.1	310	34.8	
41–59	181	32.3	95	28.8	276	31.0	
≥60	51	9.1	160	48.5	211	23.7	
**Risk factor**							
Corticosteroids/other immunosuppressive drugs	48	8.6	79	23.9	127	14.8	<0.0001
Chronic kidney failure	12	2.1	59	17.9	71	8.2	<0.0001
Diabetes	11	2.0	51	15.5	62	7.2	<0.0001
Autoimmune disease	7	1.2	43	13.0	50	5.8	<0.0001
Solid tumor	6	1.1	38	11.5	44	5.1	<0.0001
Transplantation	0	0.0	28	8.5	28	3.3	<0.0001
Cirrhosis	8	1.4	12	3.6	20	2.3	<0.05
Hematologic neoplasms	3	0.5	15	4.5	18	2.1	<0.0001
Others *	2	0.4	2	0.6	4	0.4	NA
Unknown or no risk factor	0	0.0	141	42.7	141	16.4	<0.0001
**Clinical presentation**							
CNS	390	69.5	164	49.7	554	62.2	<0.0001
Disseminated	157	28.0	120	36.4	277	31.1	<0.004
Pulmonary	10	1.8	36	10.9	46	5.1	<0.0001
Other **	4	0.7	10	3.0	14	1.6	NA
**Symptoms/signs**							
Headache	381	69.5	164	49.7	554	62.2	<0.0001
Fever	252	44.9	117	35.4	369	41.4	0.002
Confusion	238	42.4	117	35.4	355	39.8	0.02
Nausea/vomiting	244	43.5	99	30.0	343	38.5	<0.0001
Cough	133	23.7	55	16.7	188	21.1	0.006
Intracranial hypertension without hydrocephalus	111	19.8	45	13.6	156	17.5	0.009
Seizures	101	18.0	34	10.3	135	15.2	0.001
Neck stiffness/meningeal signs	81	14.4	25	7.6	106	11.9	0.001
Others ***	195	34.8	78	23.6	273	30.6	NA
**Treatment**							
AMB	74	20.7	43	28.5	117	23.0	
AMB combined	248	69.3	90	59.6	338	66.4	
Others ****	36	10.0	18	11.9	54	10.6	
**Total**	**358**	**100.0**	**151**	**100.0**	**509**	**100.0**	
**Mortality**							
	109	19.4	72	21.8	181	20.3	0.19
**Etiological agent**							
*C. neoformans*	511	97.1	268	86.2	**779**	93.1	<0.0001
*C. gattii*	15	2.9	43	13.8	**58**	6.9	<0.0001
**Total**	**526**	**62.8**	**311**	**37.2**	**837**	**93.9**	
**Molecular types**							
VNI	69	90.8	173	93.0	242	92.4	0.2
VNII	7	9.2	10	5.4	17	6.5	
VNIII			3	1.6	3	1.1	
**Total**	**76**		**186**		**262**	**100.0**	
VGI	5	35.7	14	35.0	19	35.2	
VGII	6	42.9	19	47.5	25	45.3	
VGIII	3	21.4	7	17.5	10	18.5	
**Total**	**14**		**40**		**54**	100.0	

* Risk factors: idiopathic CD4 lymphocytopenia (2), sarcoidosis (1) and alcoholism (1). ** Clinical presentation: skeletal 1, cutaneous 2, lymph node 1, peritoneal 1, unspecified 9. *** Symptoms/signs: abnormal vision (76), double vision (60), focal neurological deficit (45), meningeal signs (48), hydrocephalus (44). **** Treatment fluconazole (52), flucytosine (2).

## Data Availability

The original contributions presented in this study are included in the article/supplementary material. Further inquiries can be directed to the corresponding author. The database will be provided to interested parties upon justified request.

## Supplementary Materials

The following are available online at https://www.mdpi.com/article/10.3390/jof12010067/s1. Table S1. Colombian publications on clinical cryptococcosis (1956–2024). Table S2. Colombian cryptococcosis study group. Surveillance format. Table S3. Diagnosis method and sample types of Cryptococcosis in Colombia (2017–2024). Table S4. Antifungal susceptibility values by species complex of *Cryptococcus* clinical isolates from Colombia (2017–2024). Figure S1. Number of Colombian Cryptococcosis patients living with HIV vs. HIV-negative (1997–2024). Table S5. Incidence of cryptococcosis in Colombia in patients living with HIV and HIV-negative (2017–2024). Figure S2a,b. Gender and age in Colombian Cryptococcosis patients living with HIV vs. HIV-negative (2017–2024). Table S6. Distribution of cryptococcosis cases in Colombia by residence department and by period analyzed (1997–2024). Table S7. Distribution of cryptococcosis patients living with HIV in Colombia according to age, sex and study period (1997–2024). Table S8. Risk factors associated with cryptococcosis cases in Colombia, comparison between study periods (1997–2024). Table S9. Distribution of samples processed and positive for culture, by period in patients affected by cryptococcosis in Colombia (1997–2024). Table S10. Acknowledgments. Colombian Group for the Study of Cryptococcosis (2017–2024). References [71,72] are cited in the Supplementary Materials.
