# Supplementary material for: Cryptococcosis in Colombia: Analysis of Data from Laboratory-Based Surveillance 2017–2024
Source: J Fungi (Basel). 2026 Jan 14;12(1):67. doi: 10.3390/jof12010067 (PMC12842726; doi:10.3390/jof12010067)
Supplement: Supplementary file 1 [file jof-12-00067-s001.zip › Table S1. Colombia 1956-2024.pdf]

Table S1. Colombian publications on clinical cryptococcosis (1956-2024)

1. Lozada-Ramos H, Álvarez-Payares J, Daza-Arana JE, Salas-Marín LM. Cryptococcal meningitis in an HCV-Positive and IVDU- and HIV-negative patient: A case report and literature review. Int Med Case Rep J. **2024**; 17:855-860. doi:10.2147/IMCRJ.S486119
2. **Cortés JA, Valderrama-Ríos MC, Lizarazo J, Millán-Oñate J, Díaz-Brochero C, Gómez CH, et al**. Guía de práctica clínica para el diagnóstico y tratamiento de la criptococosis en personas adultas que viven con el VIH. Infectio **2024**;28(1):45-67; doi.org/10.22354/24223794.1166.
3. Martín-Arsanios D, Calderón CM, Trujillo JC, Hernández LA, Arias JF, Santoyo N, et al. Caracterización de la criptococosis meníngea en pacientes no infectados con virus de inmunodeficiencia humana, hospitalizados en una institución de tercer nivel. Acta Med Colomb. **2024**;49. doi.org/10.36104/amc.2024.2859
4. Hurtado-Bedoya JD, Riveros-Santoya SV. Criptococosis meníngea: características y desenlace clínico en un hospital de tercer nivel de Bogotá, Colombia. Acta Neurol Colomb. **2023**;39(4):e829. doi.org/10.22379/anc.v39i4.829
5. Bracho-Navarro DF, Cardona-Mojica SM, Gómez-Ayala JA, Gómez-Contreras MC. Criptococosis meníngea en un paciente inmunocompetente. Med Int Méx. **2023**;39(3). doi.org/10.24245/mim.v39i3.5714
6. Tirado JT, Briceño OS, Ramírez C, Páez HA, Romero S. Criptococosis pulmomar resistente a anfotericina B aislada em paciente con infección por vírus de inmunodeficiencia humana: reporte de caso. Rev Colomb Neumol. **2023**;35(1):75-81. doi.org/10.30789/rcneumologia.v35.n1.2023.610
7. Contreras Torres AM. Caracterización de infecciones por *Cryptococcus* spp. en adultos atendidos en un hospital de cuarto nivel. Tesis de grado, Universidad del Rosario, **2023**.53 páginas. doi.org/10.48713/10336_38408
8. Mejía A, Morales SA, Dávila V, Venegas LC. Criptococosis diseminada en un adulto mayor inmunocompetente: a propósito de un caso clínico. Univ. Med. **2022**;63(2). doi.org/10.11144/Javeriana.umed63-3.crip
9. Peinado-Acevedo JS, Cáceres-Galvis C, Cadona-Palacio A, Arango-Viana JC, Roldán-Pérez M, Atencia-Flórez C. Criptococosis diseminada en paciente inmunocompetente imitando un cáncer del pulmón metastásico. Medicina & Laboratorio **2022**;26:81-89. doi.org/10.36384/01232576.561
10. Sánchez EG, Acosta D, Álvarez J, Sánchez G, García-Casallas J. Criptococosis diseminada por terapia biológica, se debe gestionar el riesgo. Biomédica. **2022**;42:218-23. doi.org/10.7705/biomedica.6239

11. Velasco de Azevedo-Pereira A, Martínez-Lemus JD, Giraldo-Peñuela D, Cervantes-de la Hoz F, Kreinter-Rosenbaun H, Jiménez-Monsalve CA. Rapidly progressive dementia by cryptococcal meningitis: A case report. Infectio. **2022**;26(3):380-383. doi.org/10.22354/24223794.1067
12. **Lizarazo J, Castañeda E.** Central Nervous System Cryptococcosis due to *Cryptococcus gattii* in the Tropics. Curr Trop Med Rep. **2022**;9(1):1-7. doi: 10.1007/s40475-022-00253-w.
13. Restrepo HF, Gutiérrez C, Milanés M. Criptococosis diseminada en paciente positivo para COVID-19. Repert Med Cir. **2021**;30(Supl.1):56-60. doi.org/10.31260/RepertMedCir.01217372.1209
14. Zabaleta MA, Silva AM, Zúñiga YC, Forero FA, Lemus I. Oftalmoplejía internuclear bilateral (WEBINO) en un paciente pediátrico con lupus eritematoso sistémico. Rev Chil Neuro-psiquiat. **2021**;59(2):152-158. doi.org/10.4067/s0717-92272021000200152
15. Ávila-Coy HÁ, López-Mora MJ, Bernal-Pacheco O. Criptococosis cerebral en paciente inmunocompetente: reporte de caso y revisión de la literatura. Neurol Neurocir y Psiquiat. **2021**;49(2):69-72. doi: 10.35366/103354
16. González-Clavijo AM, Bermúdez-Silva LN, Galezo-Cuevas S, Correa-Martínez V, López-Rodríguez LV, Parra-Castañeda AL, et al. Tratamiento con fludrocortisona en una paciente con cerebro perdedor de sal, asociado a meningitis por criptococosis. Rev Colomb Endocrinol Diabet Metab. **2021**;8(4):e598. doi.org/10.53853/encr.8.4.598
17. Nassar AC, Rivera NJ, Pulido MA, León LA. Criptococosis y el sistema inmune. A propósito de un caso. Revista Cuarzo **2021**;27(1):45-57. doi.org/10.26752/cuarzo.v27.n1.518
18. Torres RE, Rosselli C, Olivares O, Agudelo S, Carrillo MP, Coral VE. Coinfección por *Cryptococcus neoformans* en paciente trasplantado renal con COVID-19. Rev Colomb Nefrol. **2021**;8(2), e521.doi.org/10.22265/acnef.8.2.521
19. Guio JK, Vargas LJ, Rozo EJ, Barón JO, Pérez DF. Ictus isquémico como complicación de la meningitis por criptococosis. Ciencia e Innovación en Salud. **2021**. E116:065-070. doi.10.17081/innosa.116
20. Martín-Arsanios D, Quintero-Muñoz E, Echeverry T, Muñoz J, Bohórquez J, Mesa C, et al. Criptococosis y Linfocitopenia T CD4 idiopática: Reporte de un caso. Infectio **2021**; 25(1): 49-54.
21. Beltrán EJ, Cianci D, López L, Mendoza MD, Saavedra DF, Viera AJ. Pesudohemorragia subaracnoidea. Presentación de un caso. Rev Colomb Radiol. **2020**;31(4):5459-5461. doi.10.53903/01212095.6
22. Martínez-Rosado LL, Cardona-Arias JA. Infecciones fúngicas en un hospital público de referencia para la atención de personas con VIH/SIDA, Medellín 2013-2017. Médicas UIS. **2020**;33(2):17-24. doi.org/10.18273/revmed.v33n2-2020002

23. Cañas A, Gómez AM, Clixto CA. Criptococosis endobronquial: reporte de caso y revisión de la literatura. Univ Med. **2020**;61(1). doi.org/10.11144/Javeriana.umed61-1.crip
24. Espinosa L, Andrade R. Renal Criptococosis. N Engl J Med. **2020**;383(24):2371. doi: 10.1056/NEJMicm2007464
25. Prieto-Ortiz R, Reyes G, Carvajal G, Figueredo E. Criptococosis pulmonar en un paciente con colitis ulcerativa tratado con prednisona y azatioprina. Rev Colomb Gastroenterol. **2020**;35(4):545-550. doi.org/10.22516/25007440.429
26. Ramírez-Melo JL, Mejía LF, Puchana ME, Rojas JP. Meningoencefalitis por *Cryptococcus gattii* en un paciente pediátrico con cirrosis: Reporte de caso. Univ Salud. **2019**; 21(3):288-92. doi.org/10.22267/rus.192103.166
27. **Noguera MC, Escandón P, Arévalo M, García Y, Suárez LE, Castañeda E.** Prevalence of cryptococcosis in Atlántico, department of Colombia assessed with an active epidemiological search. Rev Soc Bras Med Trop. **2019**;52:e20180194. doi: 10.1590/0037-8682-0194-2018
28. **Noguera MC, Escandón P, Arévalo M, Piedrahita J, Castañeda E**. Fatal neurocryptococcosis in a Colombian underage patient. J Infect Dev Ctries. **2019**;13(11):1072-1075. doi: 10.3855/jidc.9946
29. **Escandón P, Lizarazo J, Agudelo CI, Castañeda E.** Cryptococcosis in Colombia: Compilation and Analysis of Data from Laboratory-Based Surveillance. J Fungi (Basel). **2018**;4(1). pii: E32. doi.10.3390/jof4010032
30. **Firacative C, Lizarazo J, Illnait-Zaragozí MT, Castañeda E; Latin American Cryptococcal Study Group**. The status of cryptococcosis in Latin America. Mem Inst Oswaldo Cruz. 2018;113(7):e170554. doi: 10.1590/0074-02760170554. Epub 2018 Apr 5. PMID: 29641639; PMCID: PMC5888000.
31. Ramírez-Ramos C, Galindo J, Correa S, Giraldo-Bahamón G, Rivera J, Solano J, et al. Meningitis criptococócica en pacientes sin infección por VIH: presentación de dos casos y revisión de la literatura. Rev Chilen Infect. **2018**;35(6):716-721. doi.org/10.4067/S0716-10182018000600716
32. Chaves KM, Hurtado DC. Infección por *Cryptococcus* en pacientes que ingresan a una institución de tercer nivel em Bogotá. Universidad El Bosque. Tesis de grado, **2018**, 53 páginas. doi:hdl.handle.net/20.500.12495/1824
33. **Noguera MC, Escandón P, Castañeda E.** Fatal *Cryptococcus gattii* genotype VGI infection in an HIV-positive patient in Barranquilla, Colombia. Rev Inst Med Trop Sao Paulo. **2017**;59:e34.doi.org/10.1590/S1678-9946201759034.
34. Lasso FA, Zamora TO, Potosí JA, Dìaz B. Cryptococcal cerebellitis in no-VIH patient. Colomb Med (Cali). **2017**; 48(2): 94-7.
35. Hurtado IC, López P, Osorio MA, López-Medina E. Criptococosis congénita en un neonato expuesto a VIH: presentación de un caso. Infectio. **2016**(2):93-96. doi.org/10.1016/j.infect.2015.05.003

36. **Noguera MC, Escandón P, Castañeda E.** Cryptococcosis in Atlántico, Colombia: an approximation of the prevalence of this mycosis and the distribution of the etiological agent in the environment. Rev Soc Bras Med Trop. **2015**;48(5):580-6. doi: 10.1590/0037-8682-0178-2015
37. Triana J, Salgado S, Becerra GP, Pulido AC, Cárdenas K, Ramírez S. Ventriculitis e hidrocefalia secundaria a criptococosis meníngea en un paciente no-VIH: reporte de un caso en el Hospital de San José en 2014. Acta Neurol Colomb. **2015**;31(1):65-70. doi.org/10.22379/2422402210
38. Ramírez S, Roa L, Triana J, Marín J, Clavijo-Prado C, Cárdenas K, et al. Criptococosis cerebral: descripción de una serie de casos con presentaciones típicas y atípicas en el Hospital Universitario San José Infantil de Bogotá. Acta Neurol Colomb. **2015**;31(2):158-66. doi.org/10.22379/2422402223
39. Salcedo JD, Vera CA, Jaramillo LF. Criptococosis: una causa de insuficiencia adrenal. Reporte de caso y revisión de la literatura. Univ. Med. **2015**;56(4):460-469. doi.org/10.11144/Javeriana.umed56-4.ccia
40. Marriaga AP, Conde R, Durán M, Giraldo A, Mugnier J, Herrera H. Criptococosis pulmonar en el paciente inmunocompetente. Rev Colomb Neumol. **2014**;26(2):92-8. doi.org/10.30789/rcneumologia.v26.n2.2014.47.
41. **Lizarazo J, Escandón P, Agudelo CI, Firacative C, Meyer W, et al**. Retrospective Study of the Epidemiology and Clinical Manifestations of *Cryptococcus gattii* infections in Colombia from 1997–2011. PLoS Negl Trop Dis. **2014**;8: e3272. doi:10.1371/journal.pntd.0003272
42. **Lizarazo J, Escandón P, Agudelo CI, Castañeda E.** Cryptococcosis in Colombian children and literature review. Mem Inst Oswaldo Cruz. 2014 Sep;109(6):797-804. doi: 10.1590/0074-0276130537
43. **Escandón P, Lizarazo J, Agudelo CI, Chiller T, Castañeda E.** Evaluation of a rapid lateral flow immunoassay for the detection of cryptococcal antigen for the early diagnosis of cryptococcosis in HIV patients in Colombia. Med Mycol. **2013**;51(7):765-8. doi: 10.3109/13693786.2013.781692
44. Zamora TO, Agredo DC, Agredo JS. Criptococosis cerebral: análisis de 12 casos y revisión de la literatura. Medicina (Bogotá). **2013**;35(2):104-122.
45. **Lizarazo J, Chaves O, Peña Y, Escandón P, Agudelo CI, Castañeda E**. Comparación de los hallazgos clínicos y de supervivencia entre pacientes VIH positivos y VIH negativos con criptococosis meníngea en un hospital del tercer nivel. Acta Med Colomb. **2012**;37:49-61.
46. **Escandón P, de Bedout C , Lizarazo J , Agudelo CI, Tobón A , Bello S , Restrepo A, Castañeda E y Grupo Colombiano para el Estudio de la Criptococosis. Cryptococcosis in Colombia**: results of the national surveillance program for the years 2006-2010. Biomédica. **2012**;32:386-98. doi.10.7705/biomedica.v32i3.707

47. Montoya C, Paulo JD, Velásquez LF. Criptococosis ocular y retinitis por citomegalovirus en paciente inmunosuprimido. Infectio. **2012**;16 (suppl 3):100-103. doi.org/10.1016/S0123-9392(12)70033-4
48. **Lizarazo J, Castañeda E.** Consideraciones sobre la criptococosis en pacientes con sida. Infectio **2012**;16(Supl 3):94-9.doi.10.1016/S0123-9392(12)70032-2
49. **Castañeda E, Lizarazo J**. Protocolo de estudio y manejo de los pacientes con criptococosis. Infectio **2012**;16 (Supl 3):123-5. Doi.10.1016/S0123-9332(12)70038-3
50. Castro-Jiménez M, Rey-Benito G, Duque-Beltrán S, Pinilla-Guevara C, Bello-Pieruccini S, Agudelo-Mahecha C, et al. Diagnóstico de micosis oportunistas en pacientes con VIH/sida: un estudio de casos en Colombia. Infectio. **2011**; 15(2): 92-97. doi.10.1016/S0123-9392(11)70748-2
51. Gómez B, Zarco LA. Criptococosis meníngea: características clínicas y de laboratorio. Acta Neurol Colomb. **2011**;27:19-27.
52. Escobar AM, Díaz R, Posada A. Criptococosis diseminada en anciano con VIH. Acta Med Colomb. **2011**;36(2):85-89.
53. **Lizarazo J, Parra E, Parada O, Castro N, Chaves O, Peña Y**. Criptococosis diseminada con compromiso esplénico y meníngeo en una paciente con sida. Acta Med Colomb. **2010**;35:31-4. doi:10.36104/amc.2010.1581
54. Mantilla JC, Cárdenas N, Hallazgos neuropatológicos de la infección por VIH-SIDA: estudio de autopsias en el Hospital Universitario de Santander, Bucaramanga, Colombia. Colomb Med. **2009**;40(4):422-31.
55. Vásquez LA, Molina V, Toro AM, Gómez CI, Ruíz AC, Arroyave JE. Criptococosis cutánea: manifestación inicial de una infección diseminada en un paciente con trasplante renal. Rev Asoc Col Dermatol. **2008**;16 (2):91-3.
56. Rodríguez DA, Delgado JM, Durant A. Criptococosis meníngea en pacientes VIH negativo: presentación de un caso y revisión de la literatura. Salud UIS. **2008**;40(1)52-58.
57. **Lizarazo J, Linares M, de Bedout C, Restrepo A, Agudelo CI, Castañeda E y Grupo Colombiano para el Estudio de la Criptococosis.** Estudio clínico y epidemiológico de la criptococosis en Colombia: resultados de nueve años de la encuesta nacional, 1997-2005. Biomédica **2007**;27:94-109. doi.org/10.7705/biomedica.v27i1.236
58. Gaviria M, Orozco B, Gómez LM, Maya C, Estrada S, Peláez LM, et al. Manifestaciones dermatológicas en pacientes con VIH. Seguimiento en 349 pacientes. Rev Asoc Col Dermatol. **2007**;15(3):203-7.
59. **Lizarazo J, Castro F, de Arco M, Chaves O, Peña Y**. Infecciones oportunistas del sistema nervioso central en pacientes con VIH atendidos en el Hospital Universitario Erasmo Meoz de Cúcuta (1995-2005). Infectio. **2006**;10:226-231.
60. **Lizarazo J, Peña Y, Chaves O, Ramírez I, Huérfano S, Castañeda E.** Linfadenitis y meningitis por *Cryptococcus neoformans* en un paciente con sida. Acta Med Colomb. **2004**;29:44-7.

61. De Vivero A, Sossa MP, Ojeda P, Carrillo J. Criptococosis pulmonar: revisión de 20 años de experiencia Hospital San Clara E.S.E. Rev Colomb Neumol. **2004**;16(1):33-41.
62. Sandoval A, Acosta A, Rueda X, Peñaranda EO. Criptococosis sistémica en un paciente con linfoma no Hodgkin. Rev Asoc Col Dermatol. **2004**;2(3):71-74.
63. Botero JC, Ruíz J, Márquez S. Criptococosis en una paciente inmunocompetente. Informe de un caso. Revista CES Medicina. **2003**;17(1):51-56.
64. **Lizarazo J, Restrepo A, Castañeda E y el Grupo Colombiano de estudio de la criptococosis.** Supervivencia y secuelas de pacientes registrados por el Grupo Colombiano de Estudio de la Criptococosis (1997-2001). Inf Quinc Epidemiol Nac. **2002**;7(23):449-53.
65. Lizarazo J, Peña Y, Chaves O, Omaña R, Huérfano S, Castañeda E. Diagnóstico temprano de criptococosis e histoplasmosis en personas con VIH/sida, informe preliminar. Inf Quinc Epidemiol Nac **2002**;7(23):453-458.
66. Lizarazo J, Mendoza M, Palacios D, Vallejo A, Bustamante A, Ojeda E, Restrepo A, Castañeda E. Criptococosis ocasionada por *Cryptococcus neoformans* varieda*d gattii.* Acta Med Colomb. **2000**;25:171-178.
67. **Ordóñez N, Torrado E, Castañeda E**. Criptococosis meníngea de 1990 a 1995: hallazgos de laboratorio. Biomédica. **1996**;16:93-7.
68. **Lizarazo J, Rodríguez MC, Ordóñez N, Vargas JJ, Castañeda E**. Meningitis por criptococo en el Hospital Erasmo Meoz de Cúcuta. Acta Neurol Colomb. **1995**;11:259-67.
69. Álvarez MI, González LA. Criptococosis e histoplasmosis en el Hospital Universitario del Valle, Cali, Colombia. Colombia Med.**1995**;26(2):61-65
70. Vergara I, Saavedra M, Saravia J, González G, Acosta C, Pardo G. Criptococosis del sistema nervioso central. Estudio de 32 casos. Acta Med Colomb. **1993**;18(4):199-210.
71. **López S, Ordóñez N, Castañeda E**. Criptococosis con manifestaciones cutáneas. Acta Med Colomb. **1993**;18(4):229-33.
72. Prada G, Torres C, Sierra. Infección por el VIH-1: análisis de 244 casos. Acta Med Colomb. **1992**:17(5):376-382.
73. Velásquez G, Betancur J, Estrada S, et al. Procesos infecciosos observados en un grupo de 193 pacientes con SIDA. Resultados de un programa cooperativo. Acta Med Colomb. **1992**: 17 (supl 4): 272. 11. Resumen 7.
74. Acosta G, Acosta JJ, Falquez A, Escorcia J. Criptococosis y embarazo. Presentación de un caso. Rev Colomb Obst Ginecol. **1991**;42(4):316-317.
75. **Arango M, Cano LE, de Bedout C, Estrada S, Gómez I, Franco L, et al.** Histoplasmosis y criptococosis diseminada en pacientes con el síndrome de inmunodeficiencia adquirida (SIDA). Acta Med Colomb. **1990**;15(2):84-91.

76. **Arango M, De Bedout C, Restrepo A**. Incremento de la criptococosis en asociación con el síndrome de inmunodeficiencia adquirida. Acta Med Colomb. **1990**; 15 (supl 4): 260. Resumen 176.
77. **Ordóñez N, Castillo J, Moreno GS**. Criptococosis: diagnóstico por el laboratorio. Biomédica **1987**; 7: 37-41. doi.org/10.7705/biomedica.v7i1-2.1933.
78. **Ordóñez N, Castañeda E.** Criptococosis. Estudio de catorce casos con diagnóstico comprobado por el laboratorio. Biomédica. **1981**;1:87-93. doi.10.7705/biomedica.v1i3.1788
79. Borrego C, Betancur S. Meningitis subagudas y crónicas en adultos. Estudio prospectivo de 27 casos. Trib. Méd. **1980;** 62 8 29
80. Greer DL, de Polanía LA. Criptococosis en Colombia:resumen de la literatura y presentación de doce casos en el Valle del Cauca. Colomb Med. **1977**;8(4):160-6.
81. Díaz G. Criptococosis en Santander. Trib Med. **1975**;52:A13-A20.
82. Martínez MA, Díaz GO. Criptococosis diseminada con glomerulitis necrotizante. Tribuna Méd. **1975**;52:A27-A32.
83. Molina J, Restrepo A, Pineda D, Mondragón H. Criptococosis sistémica asociada a lupus eritematoso diseminado. Ant Med. **1975**;25:165.172.
84. Toro G, Saravia J, Vergara I, Sanín L, Rodríguez G. Criptococosis del sistema nervioso central. Revisión del tema y estudio de 7 casos. Rev Fac Med UN Colomb. **1973**;39:1-13.
85. Uribe PH, Restrepo MA, Díaz GF. Estudio prospectivo clínico y microbiológico de las meningitis subagudas y crónicas. Ant Med. **1973**;23:153-164.
86. Pérez J. Criptococosis y lupus eritematoso. Presentación de un caso. Acta Med Valle. **1972**;3:115-116.
87. Vergara I, Saravia J, Toro G, Román G, Navarro L. Meningitis del adulto. Revisión clínica y patológica de 400 casos. Rev Fac Med UN Colombia. **1971**; 37:321-379.
88. Pedraza MA. Mycotic infections at autopsy. A comparative study in two University Hospitals. Am J Clin Path. **1969**; 51:470-476. doi.10.1093/ajcp/51.4.470
89. Peña CE. Deep mycotic infections in Colombia. A clinicopathologic study of 162 cases. Am J Clin Pathol. **1967**;47(4):505-20. doi: 10.1093/ajcp/47.4.505
90. López F, Bedoya V. Criptococosis. Presentación de un caso. Ant Med. **1966**;16:867-874.
91. Tobón MD. Torulosis. Presentación de un caso de autopsia. Rev HSJD (Armenia). **1966**;4:10-14.
92. Jaramillo J. Torulosis meningoencefalítica en la infancia. Presentación de un caso tratado exitosamente con anfotericina B. Rev HSJD (Armenia). **1966**;4:15-23.

93. López BH, Hurtado CH, Correa GE. Las micosis profundas en el Hospital San Juan de Dios. El Médico. **1965**;10:20-26.
94. Takano J, Cuello C, Hoffman E, Correa P. Estudio de lesiones residuales pulmonares. Rev Lat Anat Patol. **1962**; 6: 63-70.
95. Buitrago E, Gómez S. Comprobación de un caso de criptococosis. Caldas Médico. **1960**: 1: 5-16.
96. Lichtenberger E, Fajardo L. Un caso de criptococosis. Instant Med. **1956**; 21:31-33.

Publications with authors' names in bold correspond to the Colombian Cryptococcosis Study Group
