# Supplementary material for: Cryptococcosis in Colombia: Analysis of Data from Laboratory-Based Surveillance 2017–2024
Source: J Fungi (Basel). 2026 Jan 14;12(1):67. doi: 10.3390/jof12010067 (PMC12842726; doi:10.3390/jof12010067)
Supplement: Supplementary file 1 [file jof-12-00067-s001.zip › Table S10. Acknowledgments.pdf]

Table S10. Colombian Group for the Study of Cryptococcosis, 2017-2024

INS<br>Grupo de Microbiología	Carolina Duarte<br>Diana Susana Lizarazo<br>Julieth Carolina Gamba	
**The national laboratory network, and the clinicians and epidemiologists of the participating centers**		
Bogotá, Cundinamarca and Boyacá	María Isabel Medina<br>Luz Maldonado	Hospital Simón Bolívar
	Carlos Alvarez<br>Judy Andrea Puerta<br>Maritza Rojas<br>Claudia Linares<br>Nidia Torres	Hospital San Ignacio
	Claudia Clavijo	Clínica San Rafael
	Clara Luz Rico	Fundación Santafé de Bogotá
	María del Pilar López<br>Nubia Escobar	Hospital de Kennedy
	Martha Isabel Garzón	Hospital El Tunal
	Luz Mary Jimenez<br>Stella Mariño	Hospital La Victoria
	Andrea López	Hospital de Meissen
	Sandra Nuñez<br>Gloria Inés Gallo<br>Catalina Figueroa	LSP Bogotá
	Rosana Mejía	Clínica Nueva, Bogotá
	Adriana Ascencio	LSP Cundinamarca
	Mildred Torres	LSP Boyacá
Antioquia and Eje Cafetero	Myrtha Arango	Universidad de Antioquia, CIB
	Carlos Agudelo<br>Diego Molina<br>Carlos Restrepo	Hospital La María
	Alejandro Vélez<br>Carlos Ignacio Gómez	Hospital Pablo Tobón Uribe
	Mayiber Henao<br>Maryan Vásquez<br>Magda Cárdenas<br>Edna Vásquez	Clínica SaludCoop Medellín
	Ana María Restrepo<br>Marcela Gaviria	Clínica CES Medellín
	Dora Rivas,<br>Luz Marina Melquizoz<br>Victoria García	Hospital General de Medellín
	Hilda Alvarez	Hospital Marco Fidel Suárez
	Liliana Franco	Hospital San Vicente de Paul
	Walter Zea	LSP Antioquía
	Martha Cecilia Kogson<br>Gilberto Manjarrés	Hospital Santa Sofía, Caldas
	María Cristina Flórez<br>Martha Cecilia Orozco	LSP Caldas
	Jaime Vanegas<br>Myriam Gómez	Hospital Universitario San Jorge, Risaralda
	Olga Isabel Peláez	Comfamiliar, Risaralda

	María Eugenia Leal<br>Myriam Gómez	LSP Risaralda
Costa Pacífica	Nancy Villamarín<br>Viviana Rodríguez<br>Sandra Rodríguez	Hospital Universitario del Valle
	Mónica Recalde<br>Claudia Rocio Castañeda<br>Juan Diego Vélez<br>Fernando Rozo<br>Juan Carlos Alvir<br>Álvaro Iván Muñoz	Fundación Clínica Valle del Lili
	Jhon Diego Vélez<br>Angelica Aristizabal	LSP Valle
	María Inés Álvarez	Universidad del Valle
	Fabiola González	Universidad del Cauca
	Liz Betty Garcia	LSP Cauca
	Yolanda Solarte	Hospital San Pedro Pasto
	Consuelo Torres<br>Sadith Trejos	LSP Nariño
Santanderes	Saí Chinome<br>Karina Angarita	HUEM Cúcuta<br>Universidad de Santander, Cúcuta
	Maria Elsy Valencia	Hospital Universitario Santander
	Ingrid Jerez	LSP Santander
Tolima and Huila	Juliana Laverde	Universidad Surcolombiana Neiva
	María Eugenia Hernández<br>Luis Fernando Duran<br>Humberto Parra	Hospital Universitario Neiva
	Ricardo Martínez	Comfamiliar Neiva
	Luz Eneida Quintero<br>Diego Salinas<br>Oswaldo Tovar	LSP Huila
	María del Rosario Aldana	Hospital Federico Lleras Ibagué
	Luis Carlos Álvarez<br>Claudia Mejía<br>Julián Valverde	LSP Tolima
Costa Caribe	María Clara Noguera	Universidad Metropolitana Barranquilla
	Adriana Marín	Clínica del Norte Barranquilla
	Marlen Guerra<br>Ilda Jaimes<br>Diosmary Palacios	LSP Atlántico
	Katherine Hernández<br>Lilibeth Torres	LSP Cesar
	Rosalba Martínez	Hospital Rosario Pumarejo Cesar
	Aura Esnid Guevara	Fundación Oportunidad Vida Córdoba
Amazonia	Rodolfo Rendón	LSP Amazonas
	Carlos Sierra	LSP Caquetá
	Sandra Hurtado<br>Zaida Centell	Hospital de Villavicencio
	Angela Gómez,<br>Norma Pavas<br>Humberto Parra<br>Claudia Castañeda	LSP Meta
