# Supplementary material for: Cryptococcosis in Colombia: Analysis of Data from Laboratory-Based Surveillance 2017–2024
Source: J Fungi (Basel). 2026 Jan 14;12(1):67. doi: 10.3390/jof12010067 (PMC12842726; doi:10.3390/jof12010067)
Supplement: Supplementary file 1 [file jof-12-00067-s001.zip › Table S2. Surveillance format.pdf]

Encuesta epidemiológica sobre la criptococosis en Colombia

Datos del paciente

Nombres y apellidos [ ]

Sexo ◯ M ◯ F

Año de nacimiento [ ]

Número documento de identidad [ ]

Departamento de nacimiento [Otro] Otro(¿Cuál?) [ ]

Departamento de residencia [Otro] Otro(¿Cuál?) [ ]

Manifestaciones clínicas	Si	No	No dato
▪ Cefalea	◯	◯	◯
▪ Náuseas o vómito	◯	◯	◯
▪ Fiebre (temperatura ≥ 38ºC)	◯	◯	◯
▪ Confusión o cambios mentales	◯	◯	◯
▪ Hipertensión intra-craneana sin hidrocefalia<br>▪ Valor de la presión de apertura del LCR en mm: [ ]	◯	◯	◯
▪ Tos (de 3 o más días de evolución, con o sin expectoración)	◯	◯	◯
▪ Pérdida de visión	◯	◯	◯
▪ Visión doble	◯	◯	◯
▪ Rigidez nucal y signos meníngeos (Kernig y Brudzinski)	◯	◯	◯
▪ Hidrocefalia comunicante	◯	◯	◯
▪ Hidrocefalia obstructiva	◯	◯	◯
▪ Convulsiones	◯	◯	◯
▪ Pérdida de peso	◯	◯	◯
▪ Otras (especificar) [ ]	◯	◯	◯

Factores de riesgo	Si	No	No dato
▪ Infección por VIH	◯	◯	◯
▪ Número de células CD4+/mL [ ]	◯	◯	◯

▪ Uso de corticosteroides	○	○	○
▪ Enfermedad autoinmune<br>¿Cuál?	○	○	○
▪ Trasplante<br>Órgano trasplantado	○	○	○
▪ Tumor sólido<br>Tipo de tumor	○	○	○
▪ Malignidad hematológica<br>Tipo de malignidad	○	○	○
▪ Diabetes mellitus	○	○	○
▪ Cirrosis	○	○	○
▪ Falla renal crónica	○	○	○
▪ Sarcoidosis	○	○	○
▪ Otro factor	○	○	○
¿Cuál?			
▪ Sin factor de riesgo aparente	○	○	○
▪ ¿Fue la criptococosis la enfermedad que definió el sida?	○	○	○

Diagnóstico de criptococosis

Fecha ☐ **Caso nuevo** ○ **Recaída** ○

Diagnóstico por laboratorio	Positiva	Negativa	No realizada
▪ Tinta china en el LCR	○	○	○
▪ Antígeno capsular en LCR	○	○	○
LFA	○	○	○
Látex	○	○	○
▪ Antígeno capsular en suero	○	○	○
LFA	○	○	○
Látex	○	○	○
▪ Prueba molecular (Film array)	○	○	○
▪ Panel de meningitis/ encefalitis	○	○	○
Cultivo para *Cryptococcus neoformans/Cryptococcus gattii*	○	○	○
¿De que muestra? LCR ☐			
Sangre ☐			
Lavado broncoalveolar ☐			
Orina ☐			
Piel ☐			
Otro (¿Cuál?)			
▪ Identificación micro	○	○	○
▪ Identificación macro	○	○	○

▪ CGB	○	○	○
▪ MALDI-TOF	○	○	○
▪ Estudio histopatológico	○	○	○
¿De cuál órgano o tejido?	○	○	○

Nota: enviar el aislamiento y las muestras al Grupo de Microbiología del INS

**Imágenes diagnósticas**			**Normal**	**Anormal**	**No dato**
Radiografía del tórax			○	○	○
**¿Resultado anormal?**	Infiltrados alveolares	☐			
	Cavitaciones	☐			
	Derrame pleural	☐			
	Calcificaciones	☐			
	Nódulo pulmonar menos de 3 cm	☐			
	Masa pulmonar mayor o igual a 3 cm	☐			
	Otra (especificar)	☐			

**Imágenes cerebrales diagnósticas**	**Si**	**No**	**No dato**
1. TAC de cráneo	○	○	○
2. Resonancia magnética (RM) de cerebro	○	○	○

Resultados de los estudios de imágenes cerebrales

**Anormalidad**	**TAC**	**RM**	**Anormalidad**	**TAC**	**RM**
▪ Masa cerebral	☐	☐	▪ Infarto	☐	☐
▪ Cerebritis	☐	☐	▪ Atrofia cerebral	☐	☐
▪ Hidrocefalia	☐	☐	▪ Dilatación de los espacios de Virchow-Robin	☐	☐
Otra (especificar):					

Clasificación

B45.0 - J99.8 Criptococosis pulmonar	☐	B45.7 Criptococosis diseminada o generalizada	☐
B45.1 - G05.2-G02.1. Criptococosis cerebral, meningitis, meningocerebral	☐	B45.8 Criptococosis especificada NCOP	☐
B45.2 - L99.8. Criptococosis cutánea o de la piel	☐	B45.9 Criptococosis (infección)	☐
B45.3 - M90.2. Criptococosis de hueso u ósea	☐		

Tratamiento						
	Medicamentos		Dosis/ día		Tiempo	
Inducción						
Consolidación						
Mantenimiento o profilaxis secundaria						

- AmB desoxicolato
- AmB complejo lipídico
- Fluconazol
- Voriconazol
- Posaconazol
- AmB dispersión coloidal
- AmB liposomal
- 5FC

Resultado de la terapia (egreso)

☐ Éxito (vivo)

☐ Falla

☐ Recaída ______ Tiempo de recaída

☐ Muerte ______ Tiempo de muerte (*tiempo de muerte luego del diagnóstico)*

☐ **No dato**

Otras terapias

☐ **Anti-TB** ☐ **Antiretroviral** ☐ **Esteroides** ☐ **Otra(s)(¿Cuál?)** ______

Imágenes	Cargar aquí los archivos digitales
Clínicas (piel, etc)	
Laboratorio (tinta china, cultivos)	
Imagenología (radiografías, tac, RM)	
Histopatología	
Otra	

Profesionales responsables de la información

Nombre del clínico: ______ Correo electrónico ______

Nombre del bacteriólogo ______ Correo electrónico ______

Entidad de Salud ______ Ciudad ______ Departamento ______-

Teléfono ______ Fecha de envío. ______

Seguimiento para el INS

Muestra recibida		Fecha de recibido
Aislamiento	☐	
LCR	☐	
Suero	☐	

Resultado		Fecha de envío
C. neoformans	☐	
C. gattii	☐	

Comentarios
