# Supplementary material for: Cryptococcosis in Colombia: Analysis of Data from Laboratory-Based Surveillance 2017–2024
Source: J Fungi (Basel). 2026 Jan 14;12(1):67. doi: 10.3390/jof12010067 (PMC12842726; doi:10.3390/jof12010067)
Supplement: Supplementary file 1 [file jof-12-00067-s001.zip › Table S3. Laboratory Dx.pdf]

Table S3. Diagnosis method and sample types of Cryptococcosis in Colombia (2017-2024)

Diagnostic method	n	%
**Culture**	**886**	**99.3**
only culture	307	34.4
+ direct examination	245	27.5
+ antigenemia	96	10.8
+ Film Array	6	0.7
+ direct examination + antigenemia	210	23.5
+ direct examination + Film Array	10	1.1
+ direct examination + antigenemia + Film Array	7	0.8
+ antigenemia + Film Array	5	0.6
**Direct examination**	**472**	**53.0**
+ culture	245	27.5
+ culture + antigenemia	210	23.5
+ culture + Film Array	10	1.1
+ culture + antigenemia + Film Array	7	0.8
**Antigenemia** Latex **(292), LFA (26)**	**318**	**35.7**
+culture + direct examination	210	23.5
+culture	96	10.8
+culture + direct examination + Film Array	7	0.8
+culture + Film Array	5	0.6
**Film array**	**33**	**3.7**
+ CSF	24	2.7
+ CSF + Blood	2	0,2
+ Blood	2	0.2
Dx only by Film Array	5	0.6
**Sample type**		
**CSF**	**595**	**67.2**
CSF only	504	
CSF + Blood	86	
CSF + Blood + BAL	2	
CSF + BAL	2	
CSF + biopsy	1	
**Blood**	**313**	**35.4**
Blood only	221	
Blood + CSF	86	
Blood + BAL + CSF	2	
Blood + BAL	1	
Blood + urine	2	
Blood + sterile body fluids	1	

**BAL**	**34**	**3.8**
BAL only	29	
CSF + Blood + BAL	2	
+ CSF	2	
+ Blood	1	
**Biopsy (lung, skin, other)**	**17**	**1.9**
Only	16	
Brain + CSF	1	
**Other sterile body fluids***	**8**	**0.9**
Only	7	
+ Blood	1	
**Catheter**	**2**	**0.2**
**Tracheal aspirate**	**1**	**0.1**
**ND**	**12**	**1.4**

*Pleural (6), ascitic fluid, peritoneal (2)
