# Supplementary material for: Cryptococcosis in Colombia: Analysis of Data from Laboratory-Based Surveillance 2017–2024
Source: J Fungi (Basel). 2026 Jan 14;12(1):67. doi: 10.3390/jof12010067 (PMC12842726; doi:10.3390/jof12010067)
Supplement: Supplementary file 1 [file jof-12-00067-s001.zip › Table S4. Atifungals.pdf]

Table S4. Antifungal susceptibility values by species complex of Cryptococcus clinical isolates from Colombia (2017-2024)

Antimycotic µg/ml	*C. neoformans* n=180		*C. gattii* n=40	
	n	%	n	%
Amphotericin B (AMB)				
0.12	73	40.6	11	27.5
0.25	75	41.7	16	40.0
0.5	14	7.8	5	12.5
**WT**	**162**	**90%**	**32**	**80%**
1	12	6.7	7	17.5
2	5	2.8	1	2.5
4	1	0.6	-	-
**NWT**	**18**	**10%**	**8**	**20%**
Itraconazole (ITR)				
0.03	2	1.1	-	-
0.06	11	6.1	2	5.0
0.12	47	26.1	2	5.0
0.25	94	52.2	27	67.5
**WT**	**154**	**85.6%**	**31**	**77.5%**
0.5	24	13.3	8	20.0
1	2	1.1	1	2.5
**NWT**	**26**	**14.5%**	**9**	**22.5%**
Fluconazole (FLC)				
2	1	0.6	0	0
4	4	2.2	0	0
8	23	12.8	1	2.5
**WT**	**28**	**15.5%**		
16	68	37.8	10	25.0
32	61	33.9	17	42.5
64	17	9.4	9	22.5
128	4	2.2	2	5.0
256	2	1.1	1	2.5
**NWT**	**152**	**84.5%**	**40**	**100%**
Voriconazole (VRC)				
0.03	1	0.6	1	2.5
0.06	10	5.6	1	2.5
0.125	37	20.6	3	7.5
**WT**	**48**	**26.7%**	**5**	**12.5%**
0.25	75	41.7	7	17.5
0.5	46	25.6	17	42.5
1	6	3.3	10	25.0
2	2	1.1	-	-
4	1	0.6	1	2.5
8	2	1.1	-	-
**NWT**	**132**	**73.3%**	**35**	**87.5%**

Antimycotic µg/ml	*C. neoformans* n=180		*C. gattii* n=40	
	n	%	n	%
Posaconazole (POS)				
0.06	2	1.1		
0.125	9	5.0	2	5.0
**WT**	**11**	**6.1**		
0.25	52	28.9	7	17.5
**WT**			**9**	**22.5**
0.5	103	57.2	26	65.0
1	14	7.8	5	12.5
**NWT**	**169**	**93.9**	**31**	**77.5**
Flucytosine (5-FC)				
0.25	10	5.6	3	7.5
0.5	2	1.1	2	5.0
1	7	3.9	1	2.5
2	25	13.9	9	22.5
WT			**15**	**37.5**
4	41	22.8	17	42.5
8	65	36.1	7	17.5
**WT**	**150**	**83.3**		
16	27	15.0	1	2.5
32	3	1.7	-	-
**NWT**	**30**	**16.7**	**25**	**62.5**

WT: wild type; **NWT:** non wild type

WT isolates were classified according to the following Epidemiological cut-off values (ECV):

AMB, MIC values ≤ 0?5 µg/ml (*C. neoformans* and *C. gattii*)
ITR, MIC values ≤ 0?25 µg/ml (*C. neoformans* and *C. gattii*)
FLC?MIC values ≤ 8 µg/ml (*C. neoformans*) and ≤ 4 µg/ml (*C. gattii*)
VRC, MIC values ≤ 0?125 µg/ml (*C. neoformans and C. gattii*)
POS, MIC values ≤ 0?125 µg/ml (*C. neoformans*) and ≤ 0?25 µg/ml (*C. gattii)*
5FC, MIC values ≤ 8 µg/ml (*C. neoformans*) and ≤ 2 µg/ml (*C. gattii*)

References

Espinel-Ingroff A, Aller AI, Canton E, Castanon-Olivares LR, Chowdhary A, Cordoba S, et al. *Cryptococcus neoformans-Cryptococcus gattii* species complex: an international study of wild-type susceptibility endpoint distributions and epidemiological cutoff values for fluconazole, itraconazole, posaconazole, and voriconazole. Antimicrob Agents Chemother. 2012;56(11):5898–906. Epub 2012/09/06. doi: 10.1128/AAC.01115-12; PubMed Central PMCID: PMC3486550.

Espinel-Ingroff A, Chowdhary A, Cuenca-Estrella M, Fothergill A, Fuller J, Hagen F, et al. *Cryptococcus neoformans-Cryptococcus gattii* species complex: an international study of wild-type susceptibility endpoint distributions and epidemiological cutoff values for amphotericin B and flucytosine. Antimicrob Agents Chemother. 2012 Jun;56(6):3107-13. doi: 10.1128/AAC.06252-11. Epub 2012 Mar 5. PMID: 22391546; PMCID: PMC3370763.
