# Supplementary material for: Cryptococcosis in Colombia: Analysis of Data from Laboratory-Based Surveillance 2017–2024
Source: J Fungi (Basel). 2026 Jan 14;12(1):67. doi: 10.3390/jof12010067 (PMC12842726; doi:10.3390/jof12010067)
Supplement: Supplementary file 1 [file jof-12-00067-s001.zip › Table S5. VIH+ vs VIH-. Incidence.pdf]

Table S5. Incidence of cryptococcosis in Colombia in patients living with HIV and HIV negative (2017-2024)

Department of residence	Living with HIV			HIV negative			Total			Incidence ratio HIV+/ HIV-
	n	Population data 2024	Rate	n	Population data 2024	Rate	n	Population data 2024	Rate	
Bogotá/Cundinamarca	185	47096	49	108	10833019	0.12	293	10880115	0.47	408
Valle	92	22641	51	42	4559736	0.11	134	4582377	0.37	464
Norte de Santander	51	5486	116	17	1611723	0.13	68	1617209	0.53	892
Antioquia	41	33578	15	31	6615823	0.06	72	6649401	0.14	250
Atlántico	33	11409	36	8	2682256	0.04	41	2693665	0.19	900
Boyacá	21	1428	184	20	1255861	0.19	41	1257289	0.41	968
Santander	20	7021	36	22	2275187	0.12	42	2282208	0.23	300
Risaralda	18	5643	40	8	957136	0.10	26	962779	0.34	400
Nariño	14	2735	64	13	1662719	0.10	27	1665454	0.20	640
Meta	14	3567	49	7	1078465	0.08	21	1082032	0.24	613
Quindío	10	3462	36	5	549859	0.11	15	553321	0.34	327
Magdalena	8	4255	24	1	1421768	0.01	9	1426023	0.08	2400
Córdoba	9	5700	20	6	1842528	0.04	15	1842585	0.10	500
Huila	7	2988	29	2	1133923	0.02	9	1136911	0.10	1450
Bolívar	7	6800	13	2	2169226	0.01	9	2169294	0.05	1300
Cauca	6	2453	31	9	1505608	0.07	15	1508061	0.12	443
Tolima	4	3577	14	6	1350761	0.05	10	1354338	0.09	280
Cesar	3	3882	10	2	1287037	0.02	5	1290919	0.05	500
Putumayo	2	563	44	0	364320	0.00	2	364883	0.07	0
Vaupés	1	21	595	1	43167	0.28	2	43188	0.58	2125
Guaviare	1	199	63	3	88851	0.44	4	89050	0.56	143
Choco	1	612	20	0	561775	0.00	1	562387	0.02	0
Casanare	1	1245	10	2	442287	0.05	3	443532	0.08	200
La Guajira	1	1955	6	2	962112	0.02	3	964067	0.04	300
Caldas	1	3197	4	2	1018238	0.02	3	1021435	0.04	200
Caquetá	0	950	0	4	412738	0.12	4	413688	0.12	0
Sucre	0	2632	0	2	947980	0.03	2	950612	0.03	0
Amazonas	0	159	0	1	80313	0.15	1	80472	0.16	0
Arauca	0	490	0	1	290762	0.04	1	291252	0.04	0
Vichada	0	58	0	0	115138	0.00	0	115196	0	0
San Andrés y Providencia	0	61781	0	0	2682256	0.04	0	61898	0.19	0
Guainía	0	35	0	0	51971	0.00	0	52006	0	0
Venezuela	8	NA	NA	2		NA	10		NA	NA
ND	2	NA	NA	1		NA	3		NA	NA
**Total**	**561**	**185954**	**38**	**330**	**50221693**	**0.08**	**891**	**50407647**	**0.22**	**475**

*Incidence rate per 100,000 habitants
