# Supplementary material for: Cryptococcosis in Colombia: Analysis of Data from Laboratory-Based Surveillance 2017–2024
Source: J Fungi (Basel). 2026 Jan 14;12(1):67. doi: 10.3390/jof12010067 (PMC12842726; doi:10.3390/jof12010067)
Supplement: Supplementary file 1 [file jof-12-00067-s001.zip › Table S6. Residencia.pdf]

Table S6. Distribution of cryptococcosis cases in Colombia by residence department and by period analyzed (1997-2024)

**Department**	**Periods**				**Total**	
	1997-2003*	2004-2010**	2011-2016***	2017-2024		
	n				n	%
Bogotá/Cundinamarca	237	167	121	293	818	28.6
Valle	122	164	112	134	532	18.6
Antioquia	120	204	61	72	457	16.0
Norte de Santander	44	63	35	68	210	7.3
Santander	16	36	62	42	156	5.4
Atlántico	7	14	42	41	104	3.6
Risaralda	35	12	12	26	85	3.0
Boyacá	7	9	14	41	71	2.5
Cauca	10	6	28	15	59	2.1
Nariño	3	6	6	27	42	1.5
Huila	13	16	3	9	41	1.4
Meta	1	9	6	21	37	1.3
Caldas	7	22	1	3	33	1.2
Tolima	8	10	5	10	33	1.2
Cesar	0	4	21	5	30	1.0
Quindío	2	3	7	15	27	0.9
Córdoba	6	4	0	15	25	0.9
Magdalena	0	5	7	9	21	0.7
Caquetá	3	4	1	4	12	0.4
Bolívar	2	0	1	9	12	0.4
La Guajira	0	0	4	3	7	0.2
Casanare	1	1	2	3	7	0.2
Arauca	2	2	0	1	5	0.2
Sucre	0	1	2	2	5	0.2
Amazonas	1	1	1	1	4	0.1
Putumayo	0	1	1	2	4	0.1
Guaviare	0	0	0	4	4	0.1
San Andrés	1	1	1	0	3	0.1
Choco	1	0	1	1	3	0.1
Vaupés	0	0	0	2	2	0.1
Venezuela	1	0	1	10	12	0.4
SD	1	0	0	3	4	0.1
**Total**	**651**	**765**	**558**	**891**	**2865**	**100.0**

* Lizarazo J, Linares M, De Bedout C, Restrepo A, Agudelo CI, Castañeda E, Grupo Colombiano para el Estudio de la Criptococosis. Estudio clínico y epidemiológico de la criptococosis en Colombia: Resultado de nueve años de la encuesta nacional, 1997–2005. Biomédica 2007, 27, 94–109.

** Escandón P, De Bedout C, Lizarazo J, Agudelo CI, Tobón A, Bello S, Restrepo A, Castañeda E, Grupo Colombiano para el Estudio de la Criptococosis. Cryptococcosis in Colombia: Results of the national surveillance program for the years 2006–2010. Biomédica 2012, 32, 386–398

*** Escandón P, Lizarazo J, Agudelo CI, Castañeda E. Cryptococcosis in Colombia: Compilation and Analysis of Data from Laboratory-Based Surveillance. J Fungi (Basel). 2018 Mar 1;4(1). pii: E32. doi: 10.3390/jof4010032
