# Supplementary material for: Cryptococcosis in Colombia: Analysis of Data from Laboratory-Based Surveillance 2017–2024
Source: J Fungi (Basel). 2026 Jan 14;12(1):67. doi: 10.3390/jof12010067 (PMC12842726; doi:10.3390/jof12010067)
Supplement: Supplementary file 1 [file jof-12-00067-s001.zip › Table S7. Sida 1997-2024.pdf]

Table S7. Distribution of cryptococcosis patients living with HIV in Colombia according to age, sex, and study period (1997-2024)

Age Group	1997–2003*				2004–2010**				2011–2016***				2017-2024**				Total				Total*
	Male		Female		Male		Female		Male		Female		Male		Female		Male		Female		
	n	%	*n*	%	*n*	%	*n*	%	*n*	%	*n*	%	*n*	%	*n*	%	*n*	%	*n*	%	n
≤16	3	50.0	3	50.0	4	66.7	2	33.3	3	100.0	0	0.0	3	60.0	2	40.0	13	65.0	7	35.0	20
17–25	44	77.2	13	22.8	59	77.6	17	22.4	45	83.3	9	16.7	55	82.1	12	17.9	203	79.9	51	20.1	254
26–40	262	91.0	36	9.0	276	83.4	55	16.6	141	84.4	26	15.6	218	84.8	39	15.2	897	85.2	156	14.8	1053
41–59	98	93.3	7	6.7	139	84.2	26	15.8	102	83.6	20	16.4	144	79.6	37	20.4	483	84.3	90	15.7	572
≥60	12	100.0	0	0.0	19	79.2	5	20.8	23	88.5	3	11.5	39	76.5	12	23.5	93	82.3	20	17.7	113
SD	15	88.2	2	11.8	10	66.7	5	33.3	15	71.4	6	28.6	0	0	0	0	40	75.5	13	24.5	53
Total	434	87.7	61	12.3	507	82.2	110	17.8	329	83.7	64	16.3	**459**	**81.8**	**102**	**18.2**	1729	83.7	337	16.3	2065
H:M	7.1:1				4.6:1				5.1:1				**4.5:1**				5.1:1				

* Lizarazo J, Linares M, De Bedout C, Restrepo A, Agudelo CI, Castañeda E, Grupo Colombiano para el Estudio de la Criptococosis. Estudio clínico y epidemiológico de la criptococosis en Colombia: Resultado de nueve años de la encuesta nacional, 1997–2005. Biomédica 2007, 27, 94–109.

** Escandón P, De Bedout C, Lizarazo J, Agudelo CI, Tobón A, Bello S, Restrepo A, Castañeda E, Grupo Colombiano para el Estudio de la Criptococosis. Cryptococcosis in Colombia: Results of the national surveillance program for the years 2006–2010. Biomédica 2012, 32, 386–398

*** Escandón P, Lizarazo J, Agudelo CI, Castañeda E. Cryptococcosis in Colombia: Compilation and Analysis of Data from Laboratory-Based Surveillance. J Fungi (Basel). 2018 Mar 1;4(1). pii: E32. doi: 10.3390/jof4010032
