# Supplementary material for: Cryptococcosis in Colombia: Analysis of Data from Laboratory-Based Surveillance 2017–2024
Source: J Fungi (Basel). 2026 Jan 14;12(1):67. doi: 10.3390/jof12010067 (PMC12842726; doi:10.3390/jof12010067)
Supplement: Supplementary file 1 [file jof-12-00067-s001.zip › Table S8. FR 1997-2024.pdf]

Table S8. Risk factors associated with cryptococcosis cases in Colombia, comparison between study periods (1997–2024)

Risk Factor	1997–2003*	2004–2010**	2011–2016***	2017-2024	Total	
	n (%)				n	%
AIDS	495 (76.0)	617 (80.6)	393 (70.4)	561 (63.0%)	2066	72.1
Corticosteroids	19 (2.9)	32 (4.2)	22 (3.9)	127 (7.8)	200	7.0
Autoimmune disease	2 (0.3)	10 (1.3)	8 (1.4)	50 (5.6)	70	2.4
Transplantation	8 (1.2)	2 (0.3)	8 (1.4)	28 (3.1)	46	1.6
Tumor	12 (1.8)	7 (0.9)	7 (1.3)	44 (4.9)	70	2.4
Diabetes	3 (0.5)	4 (0.5)	11 (2.0)	62 (7.0)	80	2.8
Cirrhosis	1 (0.2)	2 (0.3)	1 (0.2)	20 (2.2)	24	0.8
Chronic renal failure	2 (0.3)	2 (0.3)	6 (1.1)	71 (8.0)	81	2.8
Unknown or No risk factor	88 (13.5)	80 (10.5)	80 (14.3)	141 (15.8)	389	13.3
Others	21 (3.2)	9 (1.2)	22 (3.9)	22 (2.7)	74	2.6
**Total**	**651**	**765**	**558**	**891**	**2865**	**100.0**

* Lizarazo J, Linares M, De Bedout C, Restrepo A, Agudelo CI, Castañeda E, Grupo Colombiano para el Estudio de la Criptococosis. Estudio clínico y epidemiológico de la criptococosis en Colombia: Resultado de nueve años de la encuesta nacional, 1997–2005. Biomédica 2007, 27, 94–109.

** Escandón P, De Bedout C, Lizarazo J, Agudelo CI, Tobón A, Bello S, Restrepo A, Castañeda E, Grupo Colombiano para el Estudio de la Criptococosis. Cryptococcosis in Colombia: Results of the national surveillance program for the years 2006–2010. Biomédica 2012, 32, 386–398

*** Escandón P, Lizarazo J, Agudelo CI, Castañeda E. Cryptococcosis in Colombia: Compilation and Analysis of Data from Laboratory-Based Surveillance. J Fungi (Basel). 2018 Mar 1;4(1). pii: E32. doi: 10.3390/jof4010032
