# Supplementary material for: Cryptococcosis in Colombia: Analysis of Data from Laboratory-Based Surveillance 2017–2024
Source: J Fungi (Basel). 2026 Jan 14;12(1):67. doi: 10.3390/jof12010067 (PMC12842726; doi:10.3390/jof12010067)
Supplement: Supplementary file 1 [file jof-12-00067-s001.zip › Table S9 . Dx 1997-2024.pdf]

Table S9. Distribution of samples processed and positive for culture, by period in patients affected by cryptococcosis in Colombia (1997-2024)

Type of sample	Periods				Total
	1997-2003*	2004-2010**	2011-2016***	2017-2024	
	n				
**CSF**	521	588	381	504	
CSF + Blood	30	30	42	86	
CSF + Blood + BAL	1	2	1	2	
CSF + Blood + skin	1	0	1	0	
CSF + BAL	1	6	4	2	
CSF + BAL+ urine	0	1	0	0	
CSF + BAL+ skin	1	0	0	0	
CSF + skin	1	0	0	0	
CSF + biopsy				1	
**Total**	556	627	429	595	**2207**
**Blood**	26	62	93	221	
Blood + CSF	30	30	42	86	
Blood + BAL + CSF	1	2	1	2	
Blood + BAL	0	5	3	1	
Blood + urine	0	0	0	2	
Blood + skin	0	1	0		
Blood + myeloculture	0	0	1	1	
**Total**	57	100	140	313	**610**
**BAL**	10	23	10	29	
BAL+ CSF + Blood	1	2	1		
BAL + CSF	1	6	4	2	
BAL + Blood	0	5	3	1	
BAL + skin	0	1	0	2	
**Total**	12	37	18	34	**101**
Biopsy	15	10	6	17	**48**
Other samples	5	9	8	11	**33**
**Total samples**	**612**	**738**	**551**	**886**	**2786**

CSF: cerebrospinal fluid. BAL: Broncho Alveolar Lavage

* Lizarazo J, Linares M, De Bedout C, Restrepo A, Agudelo CI, Castañeda E, Grupo Colombiano para el Estudio de la Criptococosis. Estudio clínico y epidemiológico de la criptococosis en Colombia: Resultado de nueve años de la encuesta nacional, 1997–2005. Biomédica 2007, 27, 94–109.

** Escandón P, De Bedout C, Lizarazo J, Agudelo CI, Tobón A, Bello S, Restrepo A, Castañeda E, Grupo Colombiano para el Estudio de la Criptococosis. Cryptococcosis in Colombia: Results of the national surveillance program for the years 2006–2010. Biomédica 2012, 32, 386–398

*** Escandón P, Lizarazo J, Agudelo CI, Castañeda E. Cryptococcosis in Colombia: Compilation and Analysis of Data from Laboratory-Based Surveillance. J Fungi (Basel). 2018 Mar 1;4(1). pii: E32. doi: 10.3390/jof4010032
