# Supplementary figures and images for: Cryptococcosis in Colombia: Analysis of Data from Laboratory-Based Surveillance 2017–2024
Source: J Fungi (Basel). 2026 Jan 14;12(1):67. doi: 10.3390/jof12010067 (PMC12842726; doi:10.3390/jof12010067)

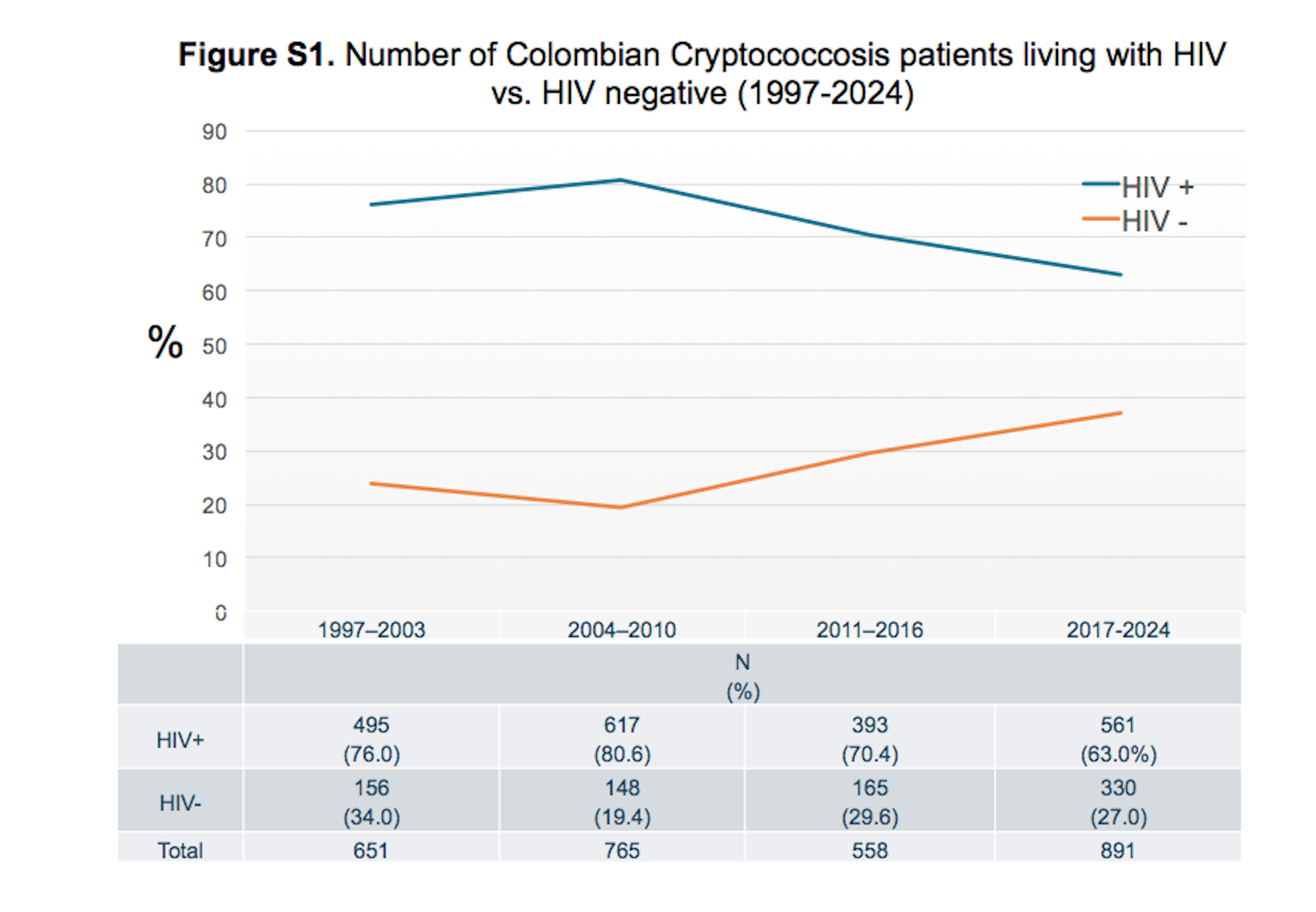

Supplement: Supplementary file 1 [file jof-12-00067-s001.zip › Figure S1.png]

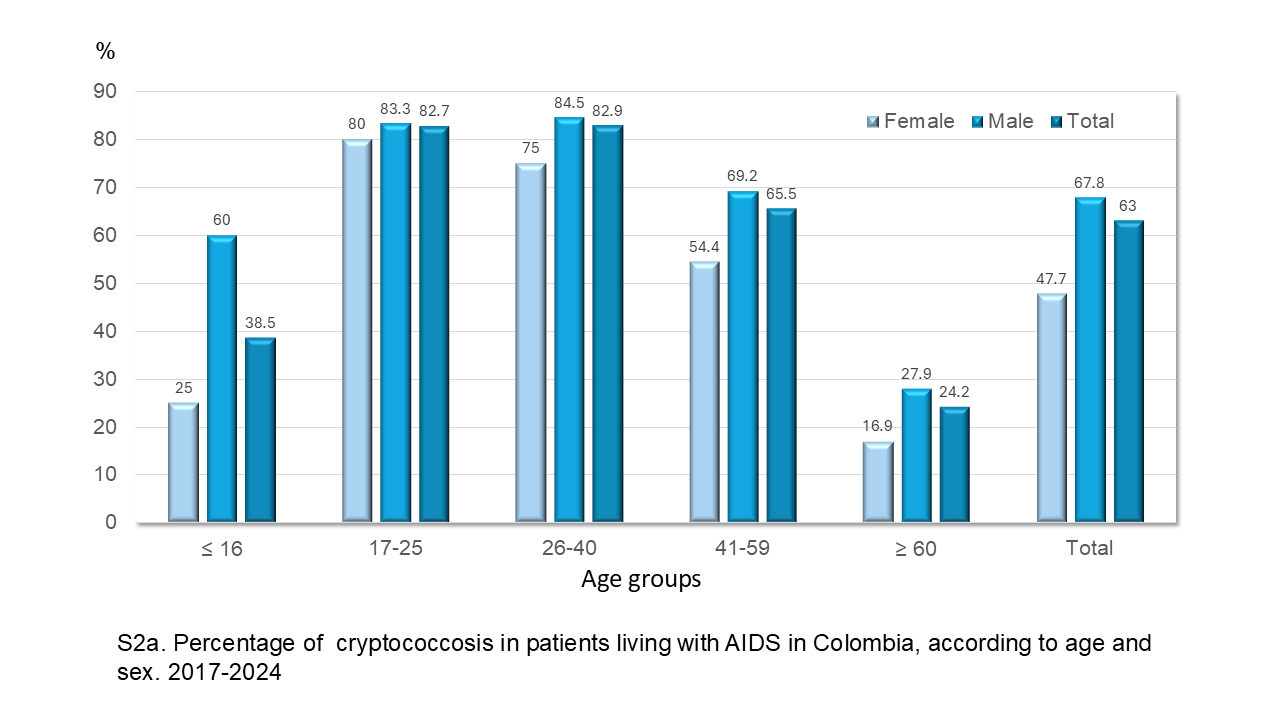

Supplement: Supplementary file 1 [file jof-12-00067-s001.zip › Figure S2a.PNG]

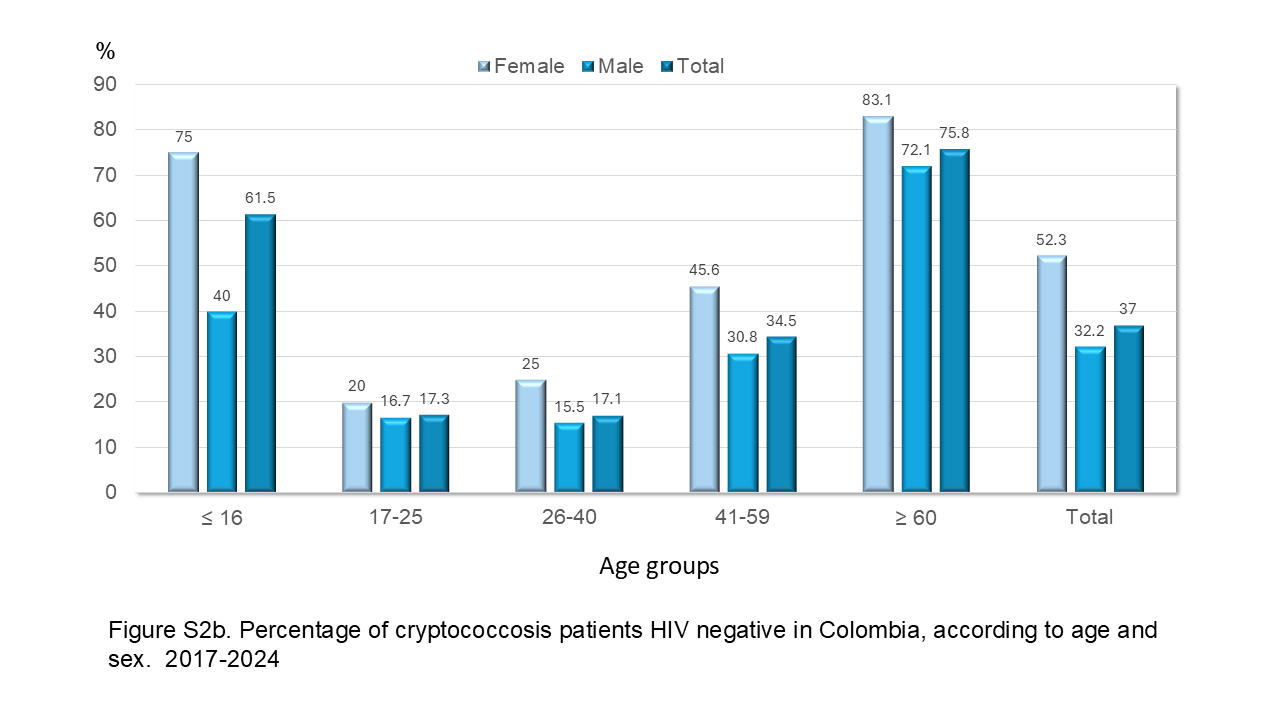

Supplement: Supplementary file 1 [file jof-12-00067-s001.zip › Figure S2b.PNG]
